# Modelling brain dynamics by Boolean networks

**DOI:** 10.1038/s41598-022-20979-x

**Published:** 2022-10-03

**Authors:** Francesca Bertacchini, Carmelo Scuro, Pietro Pantano, Eleonora Bilotta

**Affiliations:** 1 Department of Mechanics, Energy and Management Engineering, University of Calabria, Rende, Italy; 2 Laboratory of Cognitive Science and Mathematical Modelling, Department of Physics, University of Calabria, Rende, Italy; 3 Department of Physics, University of Calabria, Rende, Italy

**Keywords:** Applied mathematics, Computational science, Computational biology and bioinformatics, Neuroscience

## Abstract

Understanding the relationship between brain architecture and brain function is a central issue in neuroscience. We modeled realistic spatio-temporal patterns of brain activity on a human connectome with a Boolean networks model with the aim of computationally replicating certain cognitive functions as they emerge from the standardization of many fMRI studies, identified as patterns of human brain activity. Results from the analysis of simulation data, carried out for different parameters and initial conditions identified many possible paths in the space of parameters of these network models, with normal (ordered asymptotically constant patterns), chaotic (oscillating or disordered) but also highly organized configurations, with countless spatial–temporal patterns. We interpreted these results as routes to chaos, permanence of the systems in regimes of complexity, and ordered stationary behavior, associating these dynamics to cognitive processes. The most important result of this work is the study of emergent neural circuits, i.e., configurations of areas that synchronize over time, both locally and globally, determining the emergence of computational analogues of cognitive processes, which may or may not be similar to the functioning of biological brain. Furthermore, results put in evidence the creation of how the brain creates structures of remote communication. These structures have hierarchical organization, where each level allows for the emergence of brain organizations which behave at the next superior level. Taken together these results allow the interplay of dynamical and topological roots of the multifaceted brain dynamics to be understood.

## Introduction

Distributed networks of neuronal populations provide the foundation of behavior and cognition in animals and humans, with complex patterns of neural communication and signaling^1^. The improvements of new technologies and methods in neuroimaging have revealed a detailed structural and functional description of the human brain’s connectivity patterns^2,3^ and of its complex large-scale network. To identify the human brain as a complex dynamic system, the notion of ‘connectome’ has been proposed by Sporns et al.^4^. Since then, several investigations^5^ have employed neuro-imaging methods as well as cutting-edge graphical approaches to explore the human brain in both healthy and diseased subjects, discovering characteristic properties in different regions of the brain^3,6–9^, presenting evidence of the functional nature of clustering in both human^10,11^ and animal brains^12^, thus realizing a “comprehensive network maps of neuronal circuits and systems”^13^. The practical availability of these methods to researchers in *network neuroscience*^14^, starting from data on how brain regions are connected and adopting the theoretical framework of network science, allows the mathematical description of the brain architecture and functions. In particular, two of these mathematical organizations, small world^15^ (SWN) and scale-free^16,17^ (SF) mathematical models have been found^18^.

The approach of the small-world networks^15^ revealed spatial and topological brain architectures which allow for information travelling across the nodes of the network in all possible directions in few steps, giving rise to long distance communication and cognitive economy in healthy^19–22^ and ill subjects^23,24^, while the Barabasi and Albert model^16,17^, identified by a large variety of networks called ‘scale-free’, describes the way the brain network grows, defining the clustering organization of neuronal areas. In this kind of network, ‘preferential attachment’—gives information on the brain regions’ specialization, by a process revealing that the more a node has links, the more it will get new ones^22^. A broad range of local and global network metrics have been developed, from degree distributions, clustering, and path length to the identification of the main brain areas with their subcomponents and their spatial location, using standard anatomical atlases or random partitioning schemes to define network nodes, in order to obtain better neurobiological node delineations and thus more precise network descriptions^25,26^. In this context, structural and functional networks are of fundamental importance, where structural networks are related to patterns of anatomical links (generally synaptic links between neurons, or projections among brain areas)^4,27^, while functional networks are collected from evaluations of statistical relations between neuronal or regional time series data^28^. Most human neuroimaging studies currently employ different measures of functional connectivity^29^, but the Pearson cross-correlations of hemodynamic or electrophysiological time courses is the most used one. Instead, functional networks are highly variable, exhibiting spontaneous dynamic changes during rest^30^ as well as characteristic modulations in different task conditions^31^.

From what has been said, it follows that Connectomics not only offers a wide range of tools for brain data analysis, but it also provides an important framework to represent and interpret this “surprising machine” that is the human brain and its organization. Yet some other concepts of complexity science have not been fully exploited by networks neuroscience. Until now, well-defined networks architectures such as SWN and SF have been strongly supported by physiological evidence by real brain imaging data, the “connectome is both the source and the target of brain dynamic”^27^. But the possibility of modeling the behavior of the brain connectome by using other mathematical models is inspiring new research, considering some different points between the structural and functional connectome^32,33^. In fact, while the descriptive processes are dynamic, networks are thought to be static, and the structural connectivity is an accurate pattern of anatomical links. Instead, functional connectivity, emerging as a great set of adaptable relationships among neural elements, and historically based on the correlations of neural events recorded over time, mainly based on statistical constructs (from Granger causality to the Bayesian model), is designed to obtain information on neural interactions in neurophysiology and human imaging studies^34–37^. For this reason, it displays noteworthy variability on short and large time scales. Besides, the connectivity matrix, standing for a functional brain network, can vary according to the changing number of possible configurations of functional connections, significantly widening the number of the brain connectome underlying structural networks. Another important issue is the lack of integration of functional connectivity at different levels of neuronal organization, among different scales of the neurophysiological activity. If at the highest level of the brain scale, functional magnetic resonance imaging (fMRI) displays the activity of networks of brain regions, with their emerging population dynamics, optical imaging methods give information on the lowest level of the brain scale, with networks of individual neurons that are connected in hub regions. However, the integrated dynamics that connect the highest and the lowest scales are still unknown.

### Modelling rationale

The need to have a detailed experimental characterization of the interactional kinetics of the nodes at the brain connectome level, carefully studying the integration of both the structural and the functional connectome, at different scales of the neural organization, considering all the above-mentioned issues is gaining momentum^38–42^.

But the question of how connectivity between brain areas emerges remains underexplored. To fill this gap, a new modelling of brain networks, by using established connectomes and Boolean Networks (BN) is presented. By using the connectome as a structural network, and applying the BN approach to model the interactional dynamics of the nodes, we analyze the resulting behavior as functional neuronal dynamics. Brain dynamics can be described by many mathematical models, both discrete (such as Cellular Automata^43–47^, Boolean networks^48^, Probabilistic Boolean networks^49^) that continuous [such as Cellular Neural Networks (CNNs)^50^, Ordinary Differential Equations (ODEs) or Partial Differential Equations (PDEs)^51,52^]. We opted against using PDEs in multiple dimensions because they involve local dynamics, which are ill-suited for modeling the above-described connectome networks. By using sets of one-dimensional PDEs also introduces difficulties. While such systems efficiently describe the transmission of signals on a line, they do not provide specific information on how to explain what happens at the intersection of multiple lines, which occur as singular points. The introduction of Cellular Neural Networks (CNN) systems, by making time continuous but discretizing space, overcomes this problem, reducing network links to transmission lines^53–56^. Such modeling enables not only modeling the functioning of the brain areas of interest, which support various cognitive functions, but also to provide information about the ability to convey information across transmission lines. But, as we are interested in this work in how the process of generating cognitive circuits emerges to study the brain areas involved that give rise to specific cognitive functions, the best choice seemed that of adopting an extremely simple conceptualization such as the Boolean Networks models, which could form the baseline for further and more complex studies. Furthermore, by using BNs versus Cellular automata, one can indeed consider influences on the cognitive dynamics that are not determined by the immediate neighbors. It is obvious that thinking of the state of individual regions with only 2 values instead of a continuous state as in CNNs, or considering the system deterministic in its interactions instead of probabilistic, severely limits the possible dynamics. Despite this, the behaviors exhibited by the system, as we shall see later, maintain their complexity, typical of computational systems that, beyond the simplicity of the model, exhibit complex or chaotic behaviors and universal computational capabilities^57^. Thus, the Boolean networks model seemed to us to be the most suitable for analyzing the dynamic behavior of networks of this type without losing complexity and without introducing additional arbitrary elements into the modeling.

There are almost 6 different models of Boolean networks, which can be suitably exploited to achieve different dynamics, within the context of brain networks.

- CRBNs (Classic Random Boolean Networks), characterized by a synchronous pattern and a deterministic updating: in the passage from time $t$ to time $t + 1$ all nodes are updated simultaneously, according to the Boolean functions associated with them. These rules are not totalistic as each node evolves according to the Boolean function that characterizes it.
- ARBNs (Asynchronous Random Boolean Networks) is an asynchronous not deterministic method. At each step, a node, and only one, randomly selected, is chosen and updated with probability 1/N.
- DARBNs (Deterministic Asynchronous Random Boolean Networks) is an asynchronous and deterministic method where two parameters are assigned to each node $p$ and $q$ (after choosing a maximum value for $p$ and $q$), with $q <p$. Each node will be updated in the ${\mathrm{r }}^{\mathrm{th}}$ step if $q = r (mod p).$ If more nodes follow the updating condition in one step of evolution, they are updated one by one, taking into account the changes already made during this step.
- GARBNs (Generalized Asynchronous Random Boolean Networks) is a semi-synchronous and non-deterministic method. After choosing a $g$ number of nodes, at each step only those are updated. In this case, each node will be updated with probability equal to $g$.
- DGARBNs (Generalized Deterministic Asynchronous Random Boolean Networks) is a semi-synchronous and deterministic method. It follows the same rule DARBN, but if more nodes comply with the updating condition, they are updated simultaneously.
- PBN (Probabilistic Boolean Networks)^49^.

Consequently, in this paper we propose to model neural dynamics, maintained across a variety of different brain configuration states, at different levels of temporal and spatial scales, considering different sets of structural connectomes, from normal to many neurological disorders functioning^58^.

By using the Boolean networks approach, varying the initial conditions of the connectome pattern, we can explore how the functional interplay among brain regions operate at the macroscopic scale, with the aim to discover how the information flows in the underlying structural network, and how the dynamics arises from networks of neurons at the microscopic scale. From the simulation runs, plentiful rich dynamics emerged, giving much information on the above-mentioned issues and prospecting interesting results.

The paper is organized as follows: in Section “Methods”, we provide a review of the methods used in this work, from the approach to map on the human connectome cognitive functions related to relevant fMRI studies, to that of the implemented Boolean Network model, to the analysis of emergent circuits as large-scale structures; Section “Results” deals with the results obtained for the adopted methods; Section “Circuit analysis” considers the neuronal circuits emerging from the simulations and their correspondence to the chosen cognitive reference model; Section “Influence of the connection threshold on the stability of the emerging circuits” explores the influence of threshold parameters on the stability of emerging circuits; finally, a discussion on practical implications drawn from this approach for the network neuroscience closes the work.

## Methods

**Figure 1:**
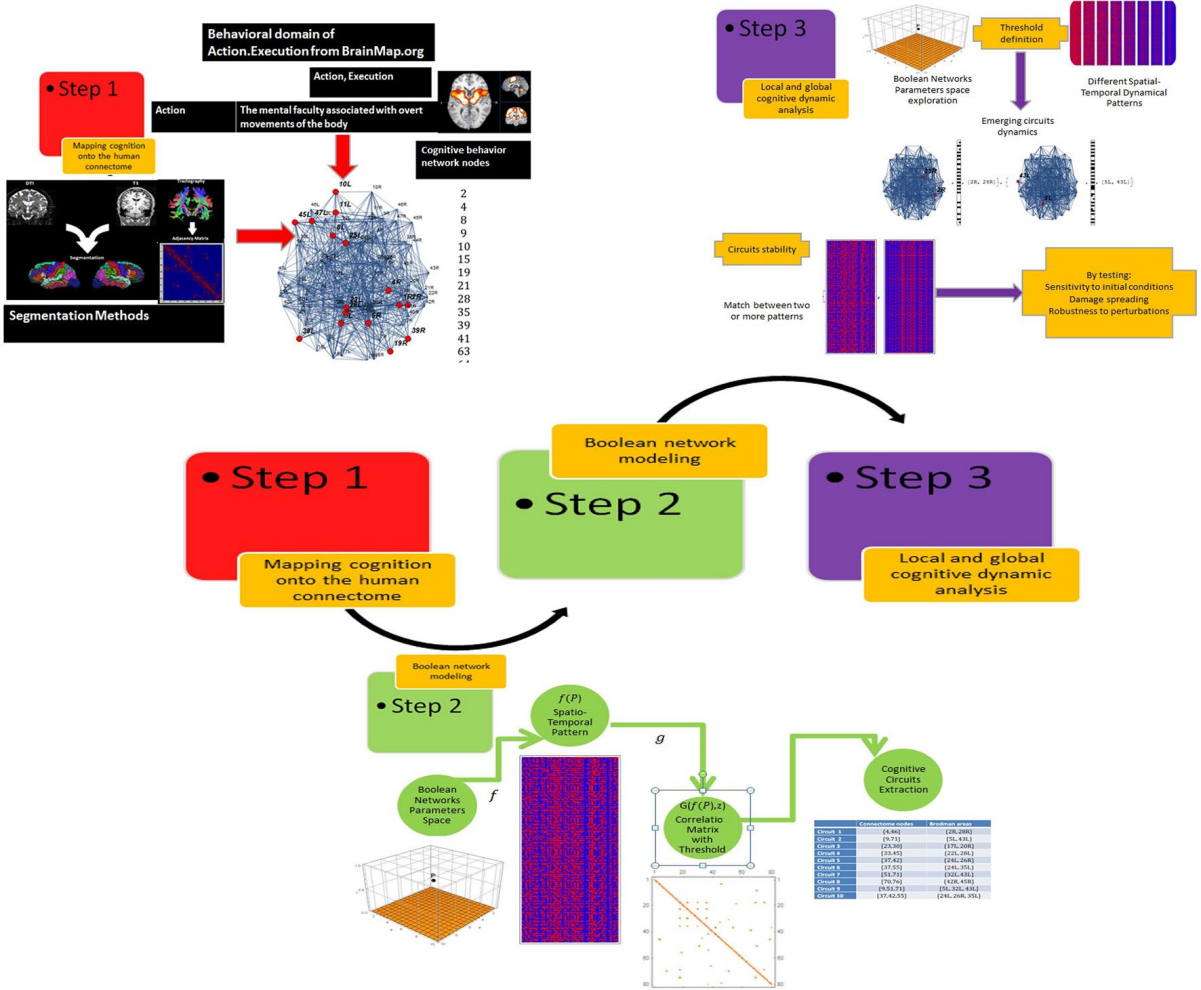
The three-step pipeline that has been used foresees the mapping of cognition onto the human connectome, the Boolean Network modeling, the circuits identification and the cognitive networks dynamical analysis at the local and the global level.

To model cognition with Boolean Networks, the methodological pipeline represented in Fig. 1 was adopted.

### Step 1: mapping cognition into the human connectome

In this paper, we simulated the brain activation points for the Action–Execution behavioral domain as identified by Smith et al.^59^ and stored in the BrainMap.org site. They were mapped on the 82 ROI connectome network (shaped on the Brodmann Atlas) and on the network developed by Mingrui et al.^60^.

Basically, we started by downloading the activation maps for the cognitive function of Action–Execution, by the behavioral taxonomy of Brainmap.org^59,61,62^, now the principal database of fMRI and PET brain activation studies. Smith et al.^41^, identified the major functional networks of the brain, representative of a significant portion of all functional activation studies performed on more than 7000 functional maps, derived from different experimental fMRI conditions, summarized in terms of coordinated positions of activation peaks, using independent component analysis (ICA), a powerful data-driven approach for finding independent patterns in multivariate data stored in the BrainMap.org data repository. We chose to simulate Action–Execution behavior, a very complex activity involving a very large neural network. After extracting the brain activation points for the Action–Execution activation map, we mapped these points with corresponding networks nodes in the brain connectome. To obtain the brain connectome^18^, we used the classic methods related to the acquisition of DTI and high-resolution T1-weighted MRI, the Segmentation of the brain’s white matter, the creation of white-matter tractography and the consequent segmentation into regions of interest (ROI), followed as standard in many software applications^63^, while the degree of an ROI allowed the definition of the role that each module plays as a source of connections with other ROI, thus improving the modelling process and the attainment of computational dynamics by using the Boolean networks^58^. In Network Neuroscience research^64^, a connectome is a mathematical representation of the brain by using nodes and edge, where each node is a region of the brain and the edges the functional links connecting the brain. The node degree corresponds to the number of edges that are attached to each node, that is the brain connectivity. Networks can be decomposed into communities or modules. Connections (edges) are either linking nodes within modules or between modules. Highly connected nodes are hubs, and they either connect primarily with other nodes in the same community (provincial hub) or with nodes that belong to different communities (connector hub). The mapping of physical and functional connections between subdivided anatomical areas of the brain with the ICAs categories of behavioral domain is a challenging problem since there are different brain parcellization methods which are related to cyto-architecture such as Brodmann^65^ and others drawn from the Talairach Atlas^66^, based on different numbers of ROIs which have been embodied in software tools^60^. Though Brodmann’s reference system of cortical maps does not match contemporary anatomical and functional data in many brain regions^67^ and new approaches are required, it is still used to register functional activations of anatomical structures^68^. For this reason, we used an 82-connectome model as the parcellization method, based on Brodmann areas, mapping the ICAs of the behavioral domain directly on the ROIs of the Brodmann organization. This allowed us to identify the network node corresponding to the functional activation areas for each category of the chosen behavioral domain, obtaining the reference areas in the 82-connectome model, involved in the process under analysis. In Table 1, Brodmann areas with the corresponding nodes of the connectome has been represented within the Tailarach space for the mapping of each node into the ICAs behavioral domain area.

**Table 1:** Table of conversion of Brodmann areas, the corresponding node number in the connectome model and the Talairach space.

Node number	Corresponding Brodmann area	Node coordinates
1	1L	{− 43.347; − 33,267; 61.135}
2	1R	{36.017; 34.877; 63.832}
3	2L	{− 46.592; − 34.283; 48.439}
4	2R	{43.042; − 34.882; 49.442}
5	3L	{− 41.124; − 26.628; 51.922}
6	3R	{38.726; − 26.779; 51.931}
7	4L	{− 27.911; − 22.877; 60.537}
8	4R	{26.884; − 22.774; 60.284}
9	5L	{− 11.711; − 50.335; 66.925}
10	5R	{10.565; − 50.376; 65.651}
11	6L	{− 28.722; − 3.387; 55.569}
12	6R	{26.821; -3.144; 55.585}
13	7L	{− 21.962; − 64.890; 53.277}
14	7R	{20.152; − 64.938; 52.170}
15	8L	{− 18.278; 22.911; 55.919}
16	8R	{16.811; 23.184; 56.088}
17	9L	{− 24.594; 37.320; 42.974}
18	9R	{22.509; 37.266; 43.076}
19	10L	{− 16.038; 59.012; 9.959}
20	10R	{13.505; 59.285; 10.292}
21	11L	{− 15.868; 41.999; − 12.318}

The table shows correspondences only up to node 21. All data on the conversion can be found at the following Internet address: https://github.com/ZhenYangCMI/BIRD_code/tree/master/dynamicAnalysis/BrainNetViewer/Data/ExampleFiles/Brodmann82. See also the SI (Table1.xls).

Areas have been connected by an adjacency matrix of 82 rows and 82 columns, whose values are comprised between 0 and 1, with an average connection value of 0.51 and a standard deviation of 0.288 for each node. This means that, on average, each network node is strongly connected with the other nodes. From a modeling point of view, since it is known that if the nodes in a network are highly connected, the system does not exhibit emergent and/or complex behaviors, going directly onto fixed-point behavior or dying immediately, we chose to set the nodes' weight values at 0.87.

### Step 2: Boolean network modelling

The concerns previously expressed about the necessity to make use of models that are explicitly defined in a computational framework is strictly tied to the requirements of accurately choosing the variables to be the most congruent possible with brain data and to explicitly and quantitatively parametrize them explicitly and quantitatively^61^. For satisfying this requirement, we have chosen a Boolean Network (BN). In the form of random Boolean Networks (RBN), the system was introduced long ago^69^, with the aim to study the regulation of gene networks. In this pioneering method, each gene had two states “0” and “1”, where the value “1” stands for the gene activation state, on the contrary the value “0” stands for an inhibition state. Since then, many studies have used this model^70^, in the sector of systems biology^71^, especially for its ability to intercept the gene networks dynamics. The status of the nodes in a Boolean network changes each time step in relation to the status of the nodes connected to it and to the Boolean function that is assigned. The number of possible states of a Boolean network, with $N$ nodes, is equal to ${2}^{N}$. This number represents the cardinality of the state space. Boolean networks can be considered synchronous or asynchronous, deterministic or non-deterministic, based on their updating rule table. In synchronous dynamics, all nodes update their status at the same time creating a "massively parallel process". In cases where the state of the system can be predictable with certainty, the dynamics of the system is deterministic. The development rules can also be totalistic in the sense that they might be the same for all nodes of the network; otherwise, they can vary according to the node. These features are very useful for sophisticated modeling of the brain cognitive functions. Formally, a Boolean network is a triplet $\{G,S,f\}$, composed of a graph $G$, a set $S=\{\mathrm{0,1}\}$ and an evolution rule $f:C\to C$, where $C$ is the space of all the possible configurations. As is well known, $G=\{V,E\}$ is made by the set $V=\{{v}_{1},{v}_{2},\cdots .{v}_{n}\}$ of $n$ elements called vertices and by the set $E=\{{\varepsilon }_{1},{\varepsilon }_{2}, \dots ,{\varepsilon }_{s}$} of any edges between two vertices ${\varepsilon }_{i}\equiv {e}_{jk}\equiv {v}_{j}\to {v}_{k}$. Each vertex ${v}_{i}$ can take a Boolean value of state ${s}_{i}=0 \, or \, {s}_{i}=1$. The critical setting of the initial state will produce different emerging configurations of the system. This is particularly suitable for simulating brain dynamics. A configuration $C=\{{s}_{1},{s}_{2},\ldots {s}_{n}\}$ is given by the values of the states of all the nodes. The cardinality of the set $C$ is equal to ${2}^{n}$. Given a node ${v}_{i}$ , the set of all nodes ${v}_{k}$ for which there is an ${\varepsilon }_{m}\in E$ such that ${e}_{ki}={v}_{k}\to {v}_{i}$ or ${e}_{ik}={v}_{i}\to {v}_{k}$ , it is said neighbor of ${v}_{i}$ , and it is indicated with ${I}_{i}$. If there is an ${e}_{m}={v}_{k}\to {v}_{i}$, then there is also an ${e}_{n}={v}_{i}\to {v}_{k}$. In this condition, we have an undirected graph or just a graph. The set of all nodes ${v}_{k}$ is called the input neighborhood and is indicated with ${I}_{i}^{i}$, while the set of all nodes ${v}_{k}$ is named output neighbor and is indicated with ${I}_{i}^{o}$, where ${I}_{i}={I}_{i}^{i}\cup {I}_{i}^{o}$. If ${I}_{i}^{i}={I}_{i}^{o}$, the graph is undirected. The cardinality of ${I}_{i}^{i}$ is called in-degree node ${v}_{i}$, and it is indicated with ${id}_{i}$ while the cardinality of ${I}_{i}^{o}$ is called out-degree node ${v}_{i}$. The Boolean network is dynamic because the configuration $c\in C$ is a function of the time. If the status of each node ${s}_{i}(t+1)$ is determined only by the value of the states of the nodes of the input neighbor ${I}_{i}^{i}$, namely:

1 $${s}_{i}(t+1)={f}_{i}({s}_{j}(t),{s}_{k}(t),\ldots ,{s}_{l}(t))$$

where each ${s}_{r}$ represents the state of the node ${{v}_{r}\in I}_{i}^{i}$, and this function does not change over time, the Boolean network is called synchronized and the dynamic system is deterministic, in the sense that the successor of a configuration is unique. Equation (1) is given usually as a table of rules ${T}_{i}$.

The evolution rule of the Boolean network $f:C\to C$ is expressed as the set $T$ of the tables of the local rules ${T}_{i}$: namely:

2 $$T=\left\{{T}_{1},{T}_{2},\ldots ,{T}_{n}\right\}$$

Since ${id}_{i}$ is the cardinality of the surrounding of ${I}_{i}^{i}$,, then the number of rules in the table of rules ${T}_{i}$ will be just ${id}_{i}$ and, therefore, the number of rules contained in the rules table of $T$ is:

$${n}_{t}=\sum_{i=1}^{n}{id}_{i}$$

In this way, all the possible combinations of inputs are equal to ${2}^{{n}_{t}}$. In the modeling of cognitive processes related to Action–Execution, the connectome with 82 nodes becomes the Boolean network. The nodes of interest are those identified for the chosen behavioral domain, whereby they will have a state of activation as the initial condition. An initial state must be set by assigning to each node a Boolean value. Later, the simulation provides for the transition of the state of each node based on the Boolean rule that is assigned to Boolean variables and the nodes to which it is connected. The state of a node at time $t$ is given by ${x}_{i}(t),$ and the next state after each iteration is given by

3 $${{x}_{i}(t + 1)=b}_{i}({x}_{i1}(t), {x}_{i2}(t) \dots {x}_{ik}(t)),$$

where ${x}_{ij}$ are the states of the nodes related to node $i$. The states of the nodes of the network are updated simultaneously according to this rule. Usually, this process is iterated until the reaching of a stable fixed point or limit cycle. This model allows a systematic exploration of the relationship between brain network structure and dynamics that might otherwise be impossible. In its most simple realization, each node of a Boolean network is connected at random to a set of K input nodes, whereas in our case the connections are provided by the connectome. In addition to the classic methods, further schemes can be introduced based on the idea of a threshold activation. According to this scheme, after choosing an initial state, the Boolean functions associated with the node will not normally be used, we instead calculate the sums of the values of the Input regions. By using this scheme, updating will take place in such a way that if at step $t$, the ${i}{\text{th}}$ node is connected to a number of a switched node that is within a given range, then it will be switched on at time $t + 1$, otherwise it will be turned off. There are many activation threshold methods, which are suitable for brain and cognitive processes networks modeling. In our case, we will use a consistent organization of the connections, linked to well-known cognitive circuits, as in^59^.

## Step 3: local and global cognitive dynamic analysis

The managing of all these variables allowed us to get data on the local interactions among brain areas.

The connections between the various Broadmann areas are given by a connection matrix 82 × 82 (see Table 1) whose values are between 0 and 1. To transform this matrix into an adjacency matrix capable of modeling the system as a not fully connected graph, it is necessary to introduce a threshold $x$ for the values of the matrix, which, below the threshold will be set equal to 0 while above the threshold will be set equal to 1.

Starting from a threshold value of 0.8, it is possible to increase this value by 0.01 every row of the matrix, thus having fluctuating values (which correspond to different values between the nodes).

If we consider the sum of states in a neighborhood ${\sigma }_{i}=\left({s}_{i1}\left(t\right)+ {s}_{i2}\left(t\right)+\dots + {s}_{ik}\left(t\right)\right)$ of the node ${v}_{i}$, then we can define the updating function (3) as follows:

4 $$\left(t + 1\right)=\left\{\begin{array}{c}1 \,  if  \, a<{\sigma }_{i}<b \\ else\\ 0\end{array}\right.$$

By varying the threshold of the $x$ matrix and the values of $a$ and $b$, we can obtain a deep investigation of brain dynamics at the global level of the systems. Once the pattern has been obtained, applying one of the above-mentioned threshold levels, we obtain a connection matrix which highlighted the emerging synchronized circuits. As we will see later, single nodes will exhibit dynamics that can be correlated with the dynamics of other nodes. By calculating these correlations and using a threshold value $z$, we could identify which dynamics have correlations above that threshold and consequently identify which nodes are correlated with each other. The method allows for the circuits extraction and identification in the network (the nodes which are connected) and in the Brodmann specifications (the involved connected Brodmann areas which have emerged from the dynamics). Many visualizations techniques, at this level, allow us to get the visualization circuit by circuit, and the overall circuit. Analysis of each circuit and of its dynamic behavior were also made. We mentioned that a Boolean network is a graph with $N$ nodes where, at each node, a Boolean variable representing the state of the node (1 or 0; ON or OFF), and a Boolean function that determines the evolution rule associated with it are assigned. To start the simulation, an initial state, assigning to each node a Boolean variable, is chosen. The dynamics provide for the transition of the state of each node based on the Boolean function that is assigned to it, and the Boolean variables of the nodes to which it is connected. So, the system behavior is emergent and unpredictable. As we have said, we will analyze both the local and the global dynamics. In local dynamics, we will investigate the interactions of each node and of its neighbors. In the global dynamics, we will study the dynamics of all the nodes that belong to the behavior under examination. This will allow us to:

- Consider the interactions of each brain region with other regions, discover the rhythms of interaction among the regions, where these exist, or if the interactions are purely random or chaotic.
- Explore the dynamics that lead to the creation of emerging networks that are structured precisely according to the process that is taking place.
- Establish an interaction time, taking care to detect whether emergencies are the result of a latent synchronization process, or whether there are submodules of the brain that synchronize and whether these submodules are in some way coordinated with each other.

Another problem we analyzed in the global dynamics behavior is the stability of the emerged circuits. Obtaining the local dynamic circuits, we try to analyze the global dynamic with an example. In an earlier evolution of BN, we got 10 circuits, considering $a=1$, $b=4$, ($x>0.9 {\text{ and}}  \, {\text{a}} \, {\text{correlation}} \, {\text{threshold z}}> 0.87))$ as network parameters and threshold values respectively. These ten circuits are considered as a reference configuration to which all the other configurations compare, obtained by changing the parameters $a$, $b$ and $x$. In this way, we identified an application in a four-dimensional space $R \times N \times N \times R$ which is the space $C$ of all the circuits. The two thresholds vary in the space $R$ of real numbers, even if the values of the connection weights matrix and the threshold values are conventionally rounded to the second decimal (varying between 0 and 1). Let us denote these discrete spaces regarding the thresholds with $H$ and $K$. Thus, we have achieved an application $f$ that, takes 4 numbers (integers—$a$ an $b$, and two numbers between 0 and 1 at the second decimal digit) as input and returns the circuits organization in the four-dimensional space:

$$g:\mathcal{P}\to C$$

where $\mathcal{P}=H \times {\mathbb{N}} \times {\mathbb{N}} \times K$ and $C$ is the set of all possible circuits sets. The problem of global stability is to understand how these circuits vary, at the variation of the 4 parameters. Setting one of the parameters ($a$, $b$, or $x$ equal to a certain value), one of the $\Gamma$ takes an equilibrium configuration ${\Gamma }_{0}$ which we call the cognitive state, assuming that this state is stable. As $a$, $b$, or $x$ can be varied, the structure deforms and assumes equilibrium configurations ${\Gamma }_{0}$ ($a$, $b$, $x,z$). These are assumed to be discretely dependent on $a$, $b$, or $x$ and stable for sufficiently small $a$, $b$, $x$ or $z$. Such a problem can be extensively studied only with a great deal of computational power. In our case, we limited our attention to the understanding of what is going on in the cognitive circuits’ stability, taking as example this first set of circuits and comparing all the circuits obtained by varying the parameters to this reference frame, changing one parameter at a time. Our hypothesis is that such circuits can vary slightly within certain limits. So, not all those that vary greatly, can be considered realistic from the point of view of their cognitive reliability. Other features that can be investigated, related to the stability of the circuits, are the concepts of “sensitivity to initial conditions”, “damage spreading”, and “robustness to perturbations”. By changing, mutating or perturbing one node or group of nodes state in the Boolean Network or by mutating their connections, or the rules of the lookup table we can quantify how a casual modification can influence the network, exploring change propagation. After discovering the circuits, we can come back to the real brain and establish whether the emerged circuits correspond to the simulated cognitive functions and whether the shortest path of the network connectome could be considered reliable for detecting how distant brain areas connect together.

## Results

Results have been organized along the pipeline of the used methods.

### Results of step 1: the structural–functional features of the network

**Figure 2:**
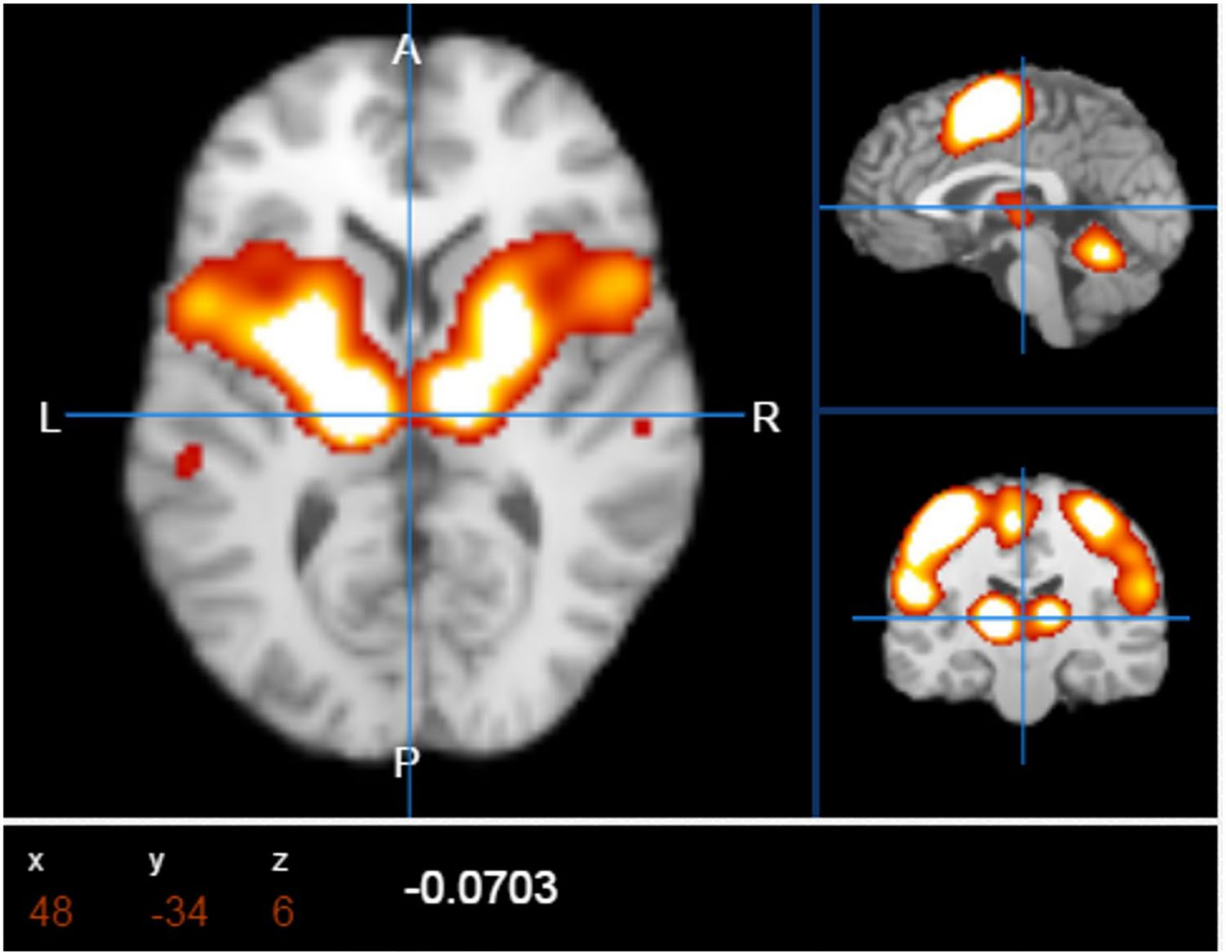
Main brain activation points for Action–Execution, archived in the BrainMap.org database. The activation points confirmed by many experimental fMRI studies are 16.

Before starting the simulation for obtaining Boolean Network simulations of the cognitive behavioral model Action–Execution (Fig. 2), the main activation map for this category was accessed at the Brain Map.org database. From the analysis of the metadata for this behavioral category, the Brodmann areas, involved in this process, were extracted and reported in Table 2. These areas, organized in the Sleuth workspace, were visualized with the software Mango, both BrainMap applications, plotting their results as coordinates on a standard brain in the Talairach space.

**Table 2:** Brodmann areas involved in the Action–Execution behavioral category.

**The somatosensory cortex**	**The primary motor cortex**
	
Brodmann areas 3, 1, and 2 refers to the primary somatosensory cortex. In the connectome, these areas correspond to nodes 1R and 2R Primary somatosensory cortex (represented by the Brodmann areas 3, 1, and 2 and corresponding to the network nodes 1R and 2R) is involved with the localization of the input stimulus, the evaluation of its intensity, the proprioception and the shape recognition processes. Area 3 receives the information that are then sent to areas 1, 2 and motor areas by the cortico-cortical neurons pathway	Brodmann area 4 denotes the primary motor cortex of the human brain. In the connectome, it corresponds to node 8. Located in the rear part of the frontal lobe, the motor cortex is involved in planning, control and execution of voluntary movements of the body, with the function of transmitting to the cells of the nuclei of the cranial nerves and cells of the spinal cord impulses for movements
**The parietal cortex**	**The frontal cortex**
	
Brodmann area 5 is part of the parietal cortex of the human brain. In the connectome, it corresponds to nodes 9 and 10. It is involved in somatosensory processing and association	Brodmann area 8 is part of the frontal cortex of the human brain. In the connectome, it corresponds to node 15. The area is involved in the management of uncertainty
**The anterior portion of the prefrontal cortex**	**The frontal cortex**
	
Brodmann area 10 is the most anterior portion of the prefrontal cortex of the human brain. In the connectome, it corresponds to node 19. Brodmann area 10, the largest area of the human brain and the most unknown, is involved in strategic processes in memory recall and various executive functions	Brodmann area 11 is part of the frontal cortex in the human brain. In the connectome, it corresponds to node 21. It is involved in decision making and processing rewards, planning, encoding new information into long-term memory, and reasoning
**The extra striate cortex**	**The posterior cingulate cortex**
	
Brodmann area 19 is part of the occipital lobe cortex in the human brain. In the connectome, it corresponds to node 28. In humans with normal sight, extrastriate cortex is a visual association area, with feature-extracting, shape recognition, attentional, and multimodal integrating functions	Brodmann area 23 corresponds to some portion of the posterior cingulate cortex. In the connectome, it corresponds to node 35. It communicates with various brain networks simultaneously and is involved in various functions such as human awareness, pain, and episodic memory retrieval
**The subgenual area of the cerebral cortex**	**The retrosplenial region of the cerebral cortex**
	
Brodmann area 25 corresponds to the subgenual area of the human cerebral cortex. In the connectome, it corresponds to node 39. Extremely rich in serotonin transporters, this area is involved in vast networks comprising areas like hypothalamus and brain stem, which affect appetite and sleep; the amygdala and insula, which affect the mood and anxiety; the hippocampus, which plays an important role in memory formation; and some parts of the frontal cortex responsible for self-esteem	Ectosplenial area 26. It is a cytoarchitecturally defined portion of the retrosplenial region of the cerebral cortex. In the connectome, it corresponds to node 41. This area is bounded externally by the granular retrolimbic area 29
**The parietal area of the cerebral cortex**	**The Broca’s area of the frontal cerebral cortex**
	
Brodmann area 39 is part of the parietal cortex in the human brain. In the connectome, it corresponds to nodes 63 and 64. Damage to Brodmann area 39 plays a role in semantic aphasia	Brodmann area 45 is part of the frontal cortex in the human brain. In the connectome, it corresponds to node 75. Together with Brodmann area 44, it comprises Broca's area, a region that is active in semantic tasks, such as semantic decision tasks (determining whether a word represents an abstract or a concrete entity) and generation tasks (generating a verb associated with a noun)
**The orbital region of the frontal cortex**	
	
Brodmann area 47 is part of the frontal cortex in the human brain. In the connectome, it corresponds to node 79. Brodmann area 47 is involved in the processing of syntax in oral and sign languages, musical syntax, and semantic aspects of language	

As we have already mentioned, such functional behavior can be accessed at the Brain Map web site (https://portal.brain-map.org/) and visualized by the Mango software application (https://ric.uthscsa.edu/mango/mango.html).

#### The structural features of the network

Standard methods were used to segment a DICOM file and get the connectome. In particular, the following procedure was conducted:

1. Acquisition of DTI and high-resolution T1-weighted MRI. Diffusion MRI allows the orientation of fibers in white and gray matter to be determined, exploiting the Brownian motion of water molecules within tissues. This motion can be isotropic—with molecules spreading in similar patterns in all directions—or anisotropic—with molecules following more irregular directional patterns. Isotropic diffusion takes a sphere-like shape, while anisotropic diffusion is ellipsoid-like.
2. Segmentation of the brain’s white matter. White Matter Tractography (WMT) includes a set of techniques that allow the connectivity and orientation of brain fibers to be estimated, through the use of images obtained by diffusion MRI. There are numerous algorithms employed, divided into deterministic, probabilistic and global optimization ones. Deterministic algorithms identify a unique trajectory from a selected starting point, and result in a path that connects discrete regions of the brain.
3. Creation of white-matter tractography. Probabilistic algorithms generate considerably more possible trajectories from a single starting point, by connecting them to several voxels and assigning to each trajectory a different probability rate. In the end, global optimization algorithms generate the optimal path between two regions of the brain minimizing a cost function, which generally describes the flow path and its goodness of fit.
4. Segmentation into regions of interest (ROI). The choice of the network nodes is made according to different atlases that allow a segmentation of the brain by different areas to be carried out (Region Of Interest). These atlases encode different areas of the brain related to specific morphological features or functionalities. Different brain segmentations in more or less defined ROIs, will yield networks with 83 or 1000 nodes using the Desikan-Killany Atlas, extended to include the subcortical regions. This is a very subtle choice, according to the aim of the analysis, as indicated in^4^. Xia et al.^72^. The lack of a universally accepted pattern for the segmentation is a well-known problem. For the purposes of this study, we chose to use 82-node networks, reported in Xia et al.^72^.
5. Construction adjacency matrix to determine the connection between the various structures. Anisotropic diffusion analysis is carried out using various methods. The most used are DTI (Diffusion Tensor Imaging) and DSI (Diffusion Spectrum Imaging). Measuring diffusivity involves the introduction of a gradient, or a variation of the magnetic field in different directions. The motion of molecules then causes changes in the intensity of the emitted signal. The gradients are applied both before and after the pulse at 180°. DTI spreading at a given point (voxel) within the brain is quantitatively described by a diffusion tensor. At least six measurements, calculated from a $3 \times 3$ symmetric matrix with six degrees of freedom, are required to construct this tensor. A connectome mapper allows the 5 steps of the procedure. It uses FREESURFER for the segmentation, FSL to display dicom files, DTK TRACKVIS to determine the tensor, and to draw the fibers and CONNECTOME to create the matrix. In the fragmentation phase, it would be possible to use the following algorithms to calculate the adjacency matrix:
  - NATIVE FREESURFER (http://surfer.nmr.mgh.harvard.edu/)^73^, (82 customized ROIs), according to Xia et al.^72^, which gives a network with 82 points, dividing the DTI and high-resolution T1-weighted MRI into 82 regions.

From a study previously carried out^74^, one sample DICOM file, from a healthy control patient, was used. The segmentations were conducted on T1 sequences, acquired by a 3 T GE, with a voxel resolution of 1 × 1 × 0.5 mm. Each slice had a 256 × 256 resolution, while total slices were 368. For the tractography process and the fibers extraction, DTI sequences were used, with a gradient number of 27 (the higher the number, the better is the resolution). The resolution of the voxel was 1 × 1 × 1. The resolution of the slice was again 256 × 256. There were 80 slices. The brain connectome was visualized by a multiplatform MATLAB (The MathWorks Inc., Natick, MA, US) toolbox, called BrainNet Viewer^72^, with an easy-to-use Graphical User Interface (GUI), which allows for the use of a visualization platform and the generation of a brain connectome with a segmentation Atlas, organized according to the Brodmann areas. Deriving many functions from the Statistical Parametric Mapping 8 application (SPM, http://www.fil.ion.ucl.ac.uk/spm/), this toolbox allows for the downloading of brain surface, node, edge and volume files. By directly downloading the ‘.pial’ file of hemisphere mesh, generated using FreeSurfer and the ‘.mesh’ files, generated using BrainVISA (http://brainvisa.info/)^75^, a connectome can be built. This tool has been chosen for the reason that it has been modeled on the automate Talairach registration procedure, developed by the Montreal Neurological Institute^76,77^, which allows the correlation between the individual volume and an average volume composed of a huge number of aligned brains to be maximized.

#### The functional features of the network

**Figure 3:**
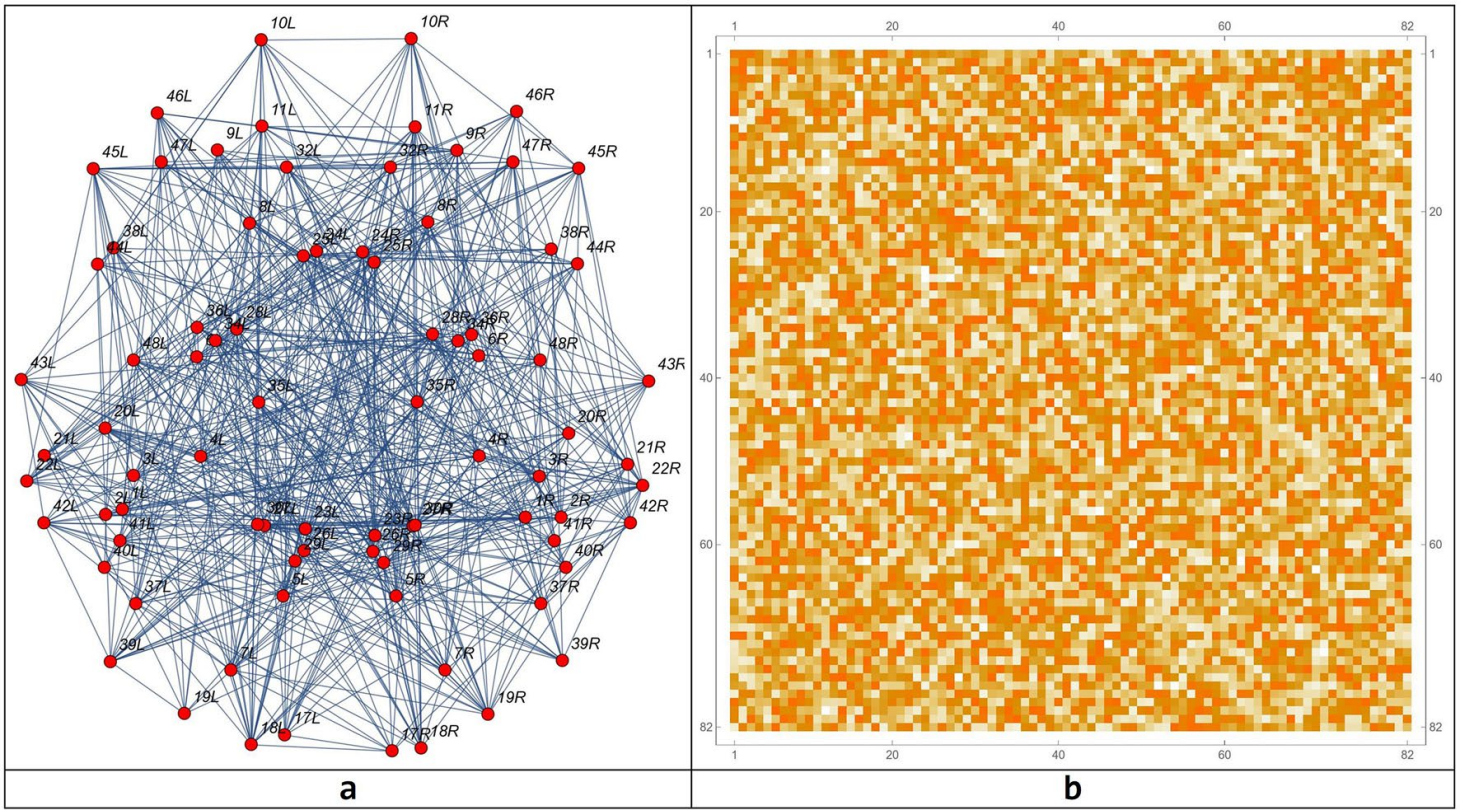
The connectome modelled on the 82 Brodmann areas (**a**) and the network connection matrix (**b**). The parcelization method adopted is as in^72^.

The transformation matrix into the Talairach space was used to define exactly the ROI related to the Action–Execution points of activation in the functional maps. After these processes, the brain connectome and its connection matrix were processed in the Mathematica environment (Fig. 3)^78^.

**Figure 4:**
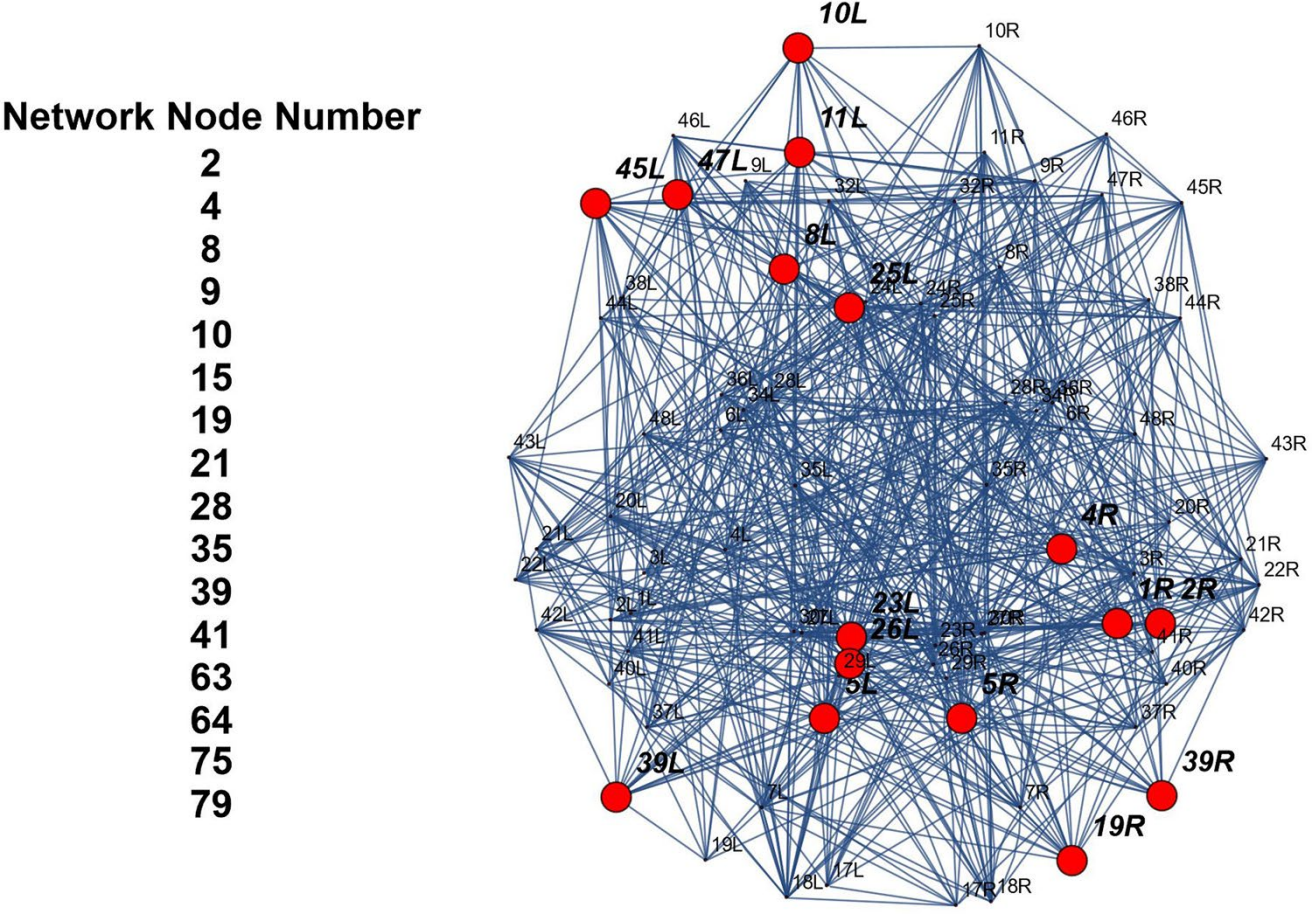
The image shows the 16 nodes of the network that produces the Action–Execution behavior.

Table 2 shows the Brodmann areas corresponding the ICAs behavioral domain area of the connectome visualized within the Talairach atlas^79^ by the Mango application software. Figure 4 shows the Action–Execution behavior from the Brodmann atlas and the corresponding node networks in the connectome.

### Results of step 2: simulation runs

In starting the simulation, we decided to activate only the 16 Brodmann areas belonging to the chosen cognitive process. Therefore, the initial condition of the Boolean network was as follows:

$$=\{\mathrm{0,1},\mathrm{0,1},\mathrm{0,0},\mathrm{0,1},\mathrm{1,1},\mathrm{0,0},\mathrm{0,0},\mathrm{1,0},\mathrm{0,0},\mathrm{1,0},\mathrm{1,0},\mathrm{0,0},\mathrm{0,0},\mathrm{0,1},\mathrm{0,0},\mathrm{0,0},\mathrm{0,0},\mathrm{1,0},\mathrm{0,0},\mathrm{1,0},\mathrm{1,0},\mathrm{0,0},\mathrm{0,0},0, \mathrm{0,0},\mathrm{0,0},\mathrm{0,0},\mathrm{0,0},\mathrm{0,0},\mathrm{0,0},\mathrm{0,0},\mathrm{0,1},\mathrm{1,0},\mathrm{0,0},\mathrm{0,0},\mathrm{0,0},\mathrm{0,0},\mathrm{0,1},\mathrm{0,0},\mathrm{0,1},\mathrm{0,0},0\};$$

Parameters of the network are as follows: $1\le a\le 13$, $1\le b\le 13$, $steps \, of \, the \,  simulation=500.$ $\mathrm{Color} \, \mathrm{Rules}->\{1\to \mathrm{Blue},0\to \mathrm{Red}\}]$.

**Figure 5:**
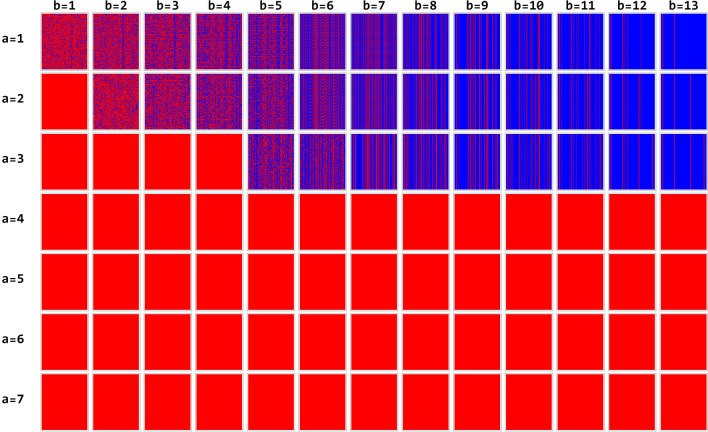
The simulation of the BN between 400 and 500 steps of simulation for values of $\mathrm{a}$ ranging from 1 to 7 and values of $\mathrm{b}$ ranging from 1 to 13.

The parameters values of the simulation offered a challenging problem as different simulations can be obtained by different network parameters. In Fig. 5, the simulation of the BN between 400 and 500 steps of simulation. At each step of the simulation, by the color rules, both activated or non-activated brain areas can be discovered. Only simulations for 1 ≤ a ≤ 7 are present in the figure, because in the other cases the system converges to a configuration for which all states go to zero.

In this simulation, there are only 91 possible rules of interest (i.e., that do not converge to a fixed point composed of only 0), concentrated for low values of a: $1\le a\le 3$.

After a transient period of 500 evolution steps, the system begins to exhibit periodic behavior. Results are reported in Table 3.

**Table 3:** Temporal emergence of periodic behavior in the brain connectome.

	b = 1	b = 2	b = 3	b = 4	b = 5	b = 6	b = 7	b = 8	b = 9	b = 10	b = 11	b = 12	b = 13
a = 1	No period	199	248	48	35	6	4	2	2	2	2	2	1
a = 2	1	No period	No period	No period	No period	6	4	2	2	2	2	2	1
a = 3	1	1	1	1	No period	158	2	2	2	2	2	2	1

**Figure 6:**
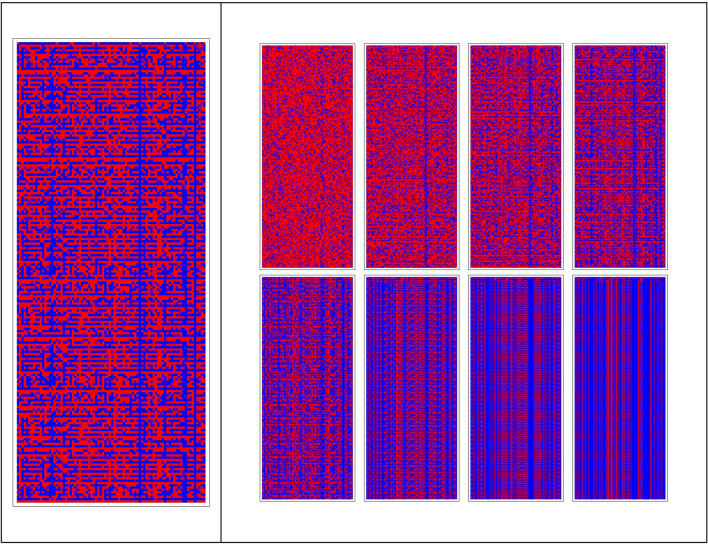
Multiple evolutions of the BN for 200 steps of simulation. This run was attained for parameter $\mathrm{b},$ going from 1 to 8, and a fixed value of $\mathrm{a}=1$. As can be seen, the patterns of the BN goes from chaos to order (from left to right), according to the increase of parameter $\mathrm{b}.$ The magnified pattern on the left is for $\mathrm{b}=4$.

We observe that for low values of $b$ the system presents chaotic (no period) or complex (high or medium periodicity) behaviors, while for high values of $b$ the system tends to have more regular periodic behaviors, with a small period. Figure 6 shows details of the spatio-temporal patterns for $a=1$ and $1\le b\le 6 .$

### Results of step 3: the emergence of brain circuits

**Figure 7:**
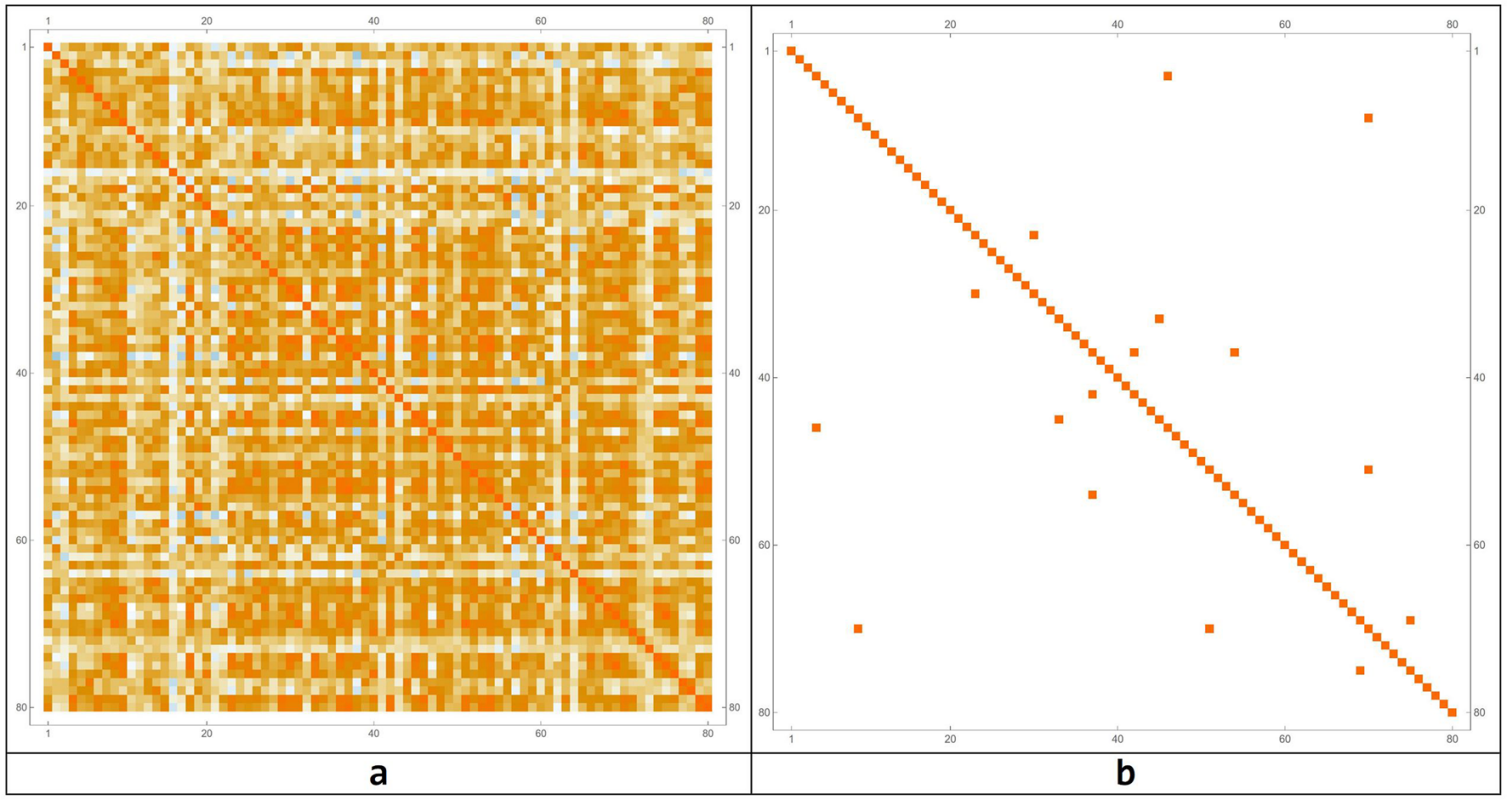
Correlation matrix among all the nodes that change over time (**a**) and with a threshold (**b**). In this case, the threshold level has been set to 0.87.

To highlight the complex emerging dynamics and the presence of structures in the simulations carried out, a model simulation ($b = 4$, network threshold x > 0.9 and, correlation threshold z > 0.87, same initial conditions), in order to consider a sufficiently complex but not chaotic behavior, is analyzed in depth. In this simulation, nodes always turned on and off have been removed, as they do not take part in the dynamics that is going on if not in a subtle way, which we will analyze in the next paragraphs. After the irrelevant nodes have been deleted, we sought correlations among those that changed over time, by means of a correlation matrix (for *b* = 4), as shown in Fig. 7.

**Figure 8:**
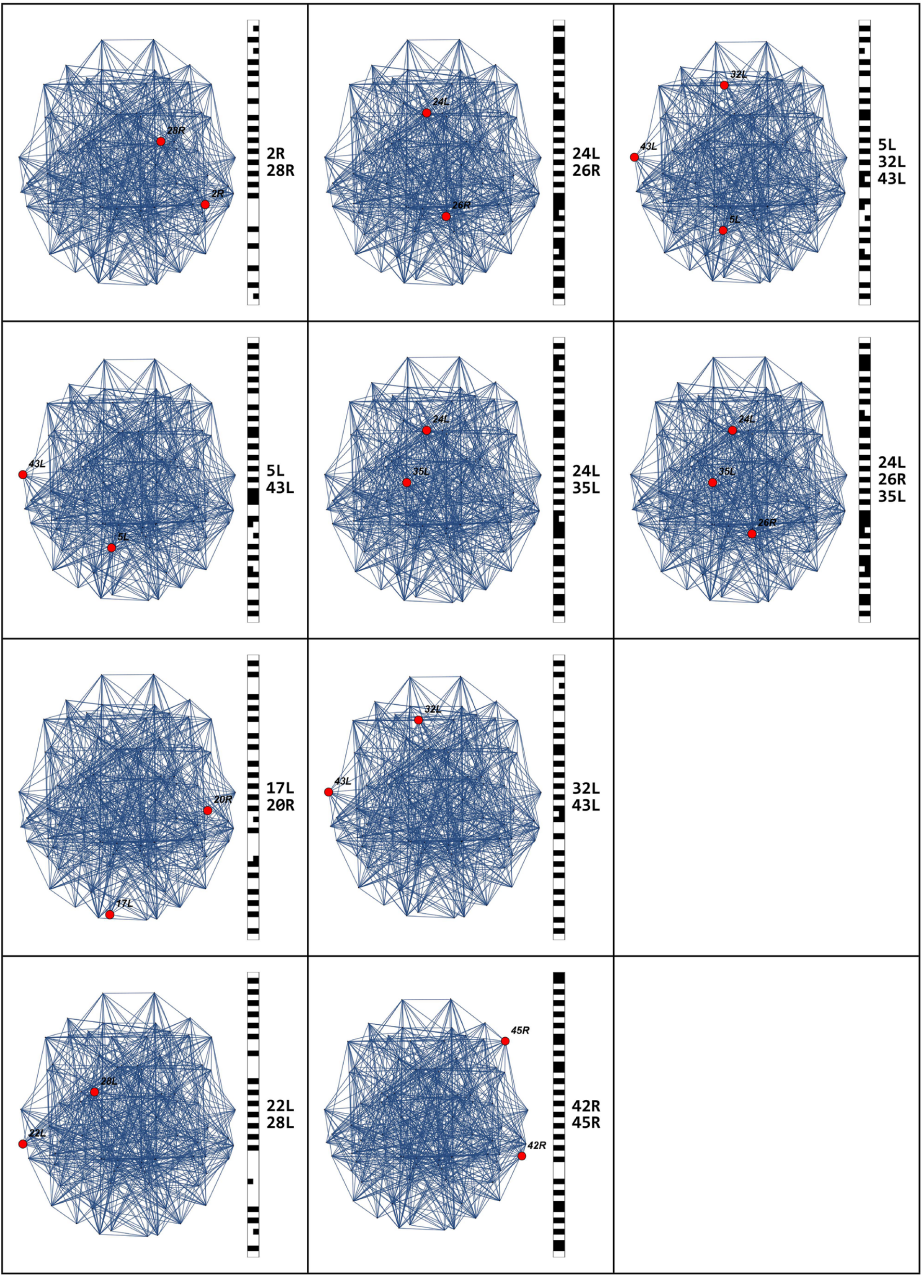
Representation of the emerged circuits in the brain connectome, setting the threshold parameter of connection among nodes at 0.87.

The correlation matrix provides us with a measure of how the performance of a brain area is related to the performance of another area. This value is between 0 and 1. When the correlation between the two areas is equal to 1, the two areas are perfectly synchronized. If we insert a $z$ threshold, selecting the pairs of areas that have a correlation higher than $z$, then we can identify groups of areas that have fairly similar behavior and we call these areas *brain circuits*. The model simulation gave 10 emerging circuits. The number of areas for each of these circuits is as follows: {2, 2, 2, 2, 2, 2, 2, 2, 3, 3}. Each circuit connects with up to three nodes of the brain connectome. In Table 4, the emerged circuits are reported, while in Fig. 8, the representation of the emerged circuits on the brain connectome is shown.

**Table 4:** Number of involved nodes and circuits for a simulation in which the threshold value was set at 0.87.

	Connectome nodes	Brodmann areas
Circuit 1	{4, 46}	{2R, 28R}
Circuit 2	{9, 71}	{5L, 43L}
Circuit 3	{23, 30}	{17L, 20R}
Circuit 4	{33, 45}	{22L, 28L}
Circuit 5	{37, 42}	{24L, 26R}
Circuit 6	{37, 55}	{24L, 35L}
Circuit 7	{51, 71}	{32L, 43L}
Circuit 8	{70, 76}	{42R, 45R}
Circuit 9	{9, 51, 71}	{5L, 32L, 43L}
Circuit 10	{37, 42, 55}	{24L, 26R, 35L}

In Table 5, results about the reliability of the emerged circuits agreeing to the cognitive behavior Action–Execution is evaluated, considering the ROIs normally used in the previously-mentioned real process.

**Table 5:** Evaluation results about the emerging circuits for the simulation chosen as example.

	Connectome nodes	Brodmann areas	Cognitive functions	Evaluation with the real process
Circuit 1	$\{\mathrm{4,46}\}$	$\left\{2\mathrm{R},28\mathrm{R}\right\}$ The Primary Somatosensory Cortex bound with the Entorhinal cortex	Area 2 analyzes size and shape of stimuli Area 28R identifies the space configuration	Believable
Circuit 2	$\{\mathrm{9,71}\}$	$\left\{5\mathrm{L},43\mathrm{L}\right\}$ The Somatosensory Association Cortex bound with the Primary gustatory cortex	Area 5 integrates sensorial stimuli Area 43 is the primary gustatory cortex, but it is active also in semantic tasks	Unbelievable, it could be positive if Area 5 is connected to areas 41 and 42 (Auditory cortex)
Circuit 3	$\{\mathrm{23,30}\}$	$\left\{17\mathrm{L},20\mathrm{R}\right\}$ The Primary visual cortex (V1) bound with the Inferior temporal gyrus	Area 17 processes the visual information Area 20 is involved in the recognition of complex object features, face perception, numbers	Believable
Circuit 4	$\{\mathrm{33,45}\}$	$\left\{22\mathrm{L},28\mathrm{L}\right\}$ The Superior temporal gyrus bound with the Ventral entorhinal cortex	Area 22 is involved in speech processing Area 28, near the hippocampus, is related to spatial memories	Believable
Circuit 5	$\{\mathrm{37,42}\}$	$\left\{24\mathrm{L},26\mathrm{R}\right\}$ The Ventral anterior cingulate cortex bound with the Retrosplenial region of the cerebral cortex	Area 24 is related to emotion formation and processing, learning, and memory Area 26 plays a probable role in mediating between perceptual and memory functions	Believable
Circuit 6	$\{\mathrm{37,55}\}$	$\{24\mathrm{L},35\mathrm{L}\}$ The Ventral anterior cingulate cortex bound with the Perirhinal cortex	Area 24 is related to emotion formation and processing, learning, and memory Area 35 is heavily related to memory processes	Believable
Circuit 7	$\{\mathrm{51,71}\}$	$\{32\mathrm{L},43\mathrm{L}\}$ The Dorsal anterior cingulate cortex bound with the Primary gustatory cortex (probably 41 and 42)	Area 32 is involved in rational thought processes Area 43 is the primary gustatory cortex, but it is active also in semantic tasks	Unbelievable
Circuit 8	$\{\mathrm{70,76}\}$	$\{42\mathrm{R},45\mathrm{R}\}$ The Auditory cortex bound with the Pars triangularis with part of the inferior frontal gyrus and part of the Broca's area	Area 42 receives input from the ears, transmits signals back, connects with other parts of the cerebral cortex Area 45 is related to semantic decision tasks (abstract or concrete nouns discrimination) generation task (generation of a verb associated with a noun)	Believable
Circuit 9	$\{\mathrm{9,51,71}\}$	$\left\{5\mathrm{L},32\mathrm{L},43\mathrm{L}\right\}$ The Somatosensory Association Cortex bound with the Dorsal anterior cingulate cortex, bound with the Primary gustatory cortex	Area 5 integrates sensorial stimuli Area 32 is involved in rational thought processes Area 43 is the primary gustatory cortex, but it is active also in semantic tasks	Unbelievable
Circuit 10	$\{\mathrm{37,42,55}\}$	$\{24\mathrm{L},26\mathrm{R},35\mathrm{L}\}$ The Ventral anterior cingulate cortex bound with the Ectosplenial portion of the retrosplenial region of the cerebral cortex bound with the Perirhinal cortex	Area 24 is related to emotion formation and processing, learning, and memory Area 26 plays a probable role in mediating between perceptual and memory functions Area 35 is heavily related to memory processes	Believable

It is reasonable to think that most of the circuits are congruent with the simulated cognitive behavior.

## Circuit analysis

Let us investigate whether there is any relationship among the emerged circuits for this simulation. The easiest and quickest way is to adopt the intersections between the areas of the emerged circuits. In Table 6 we show the intersection between these 10 circuits.

**Table 6:**
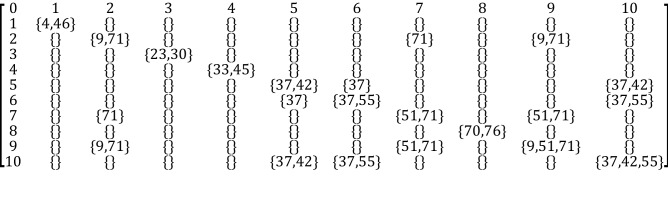
Diagram of representation of the intersection among the emerged circuits for the simulation takes as example.

**Figure 9:**
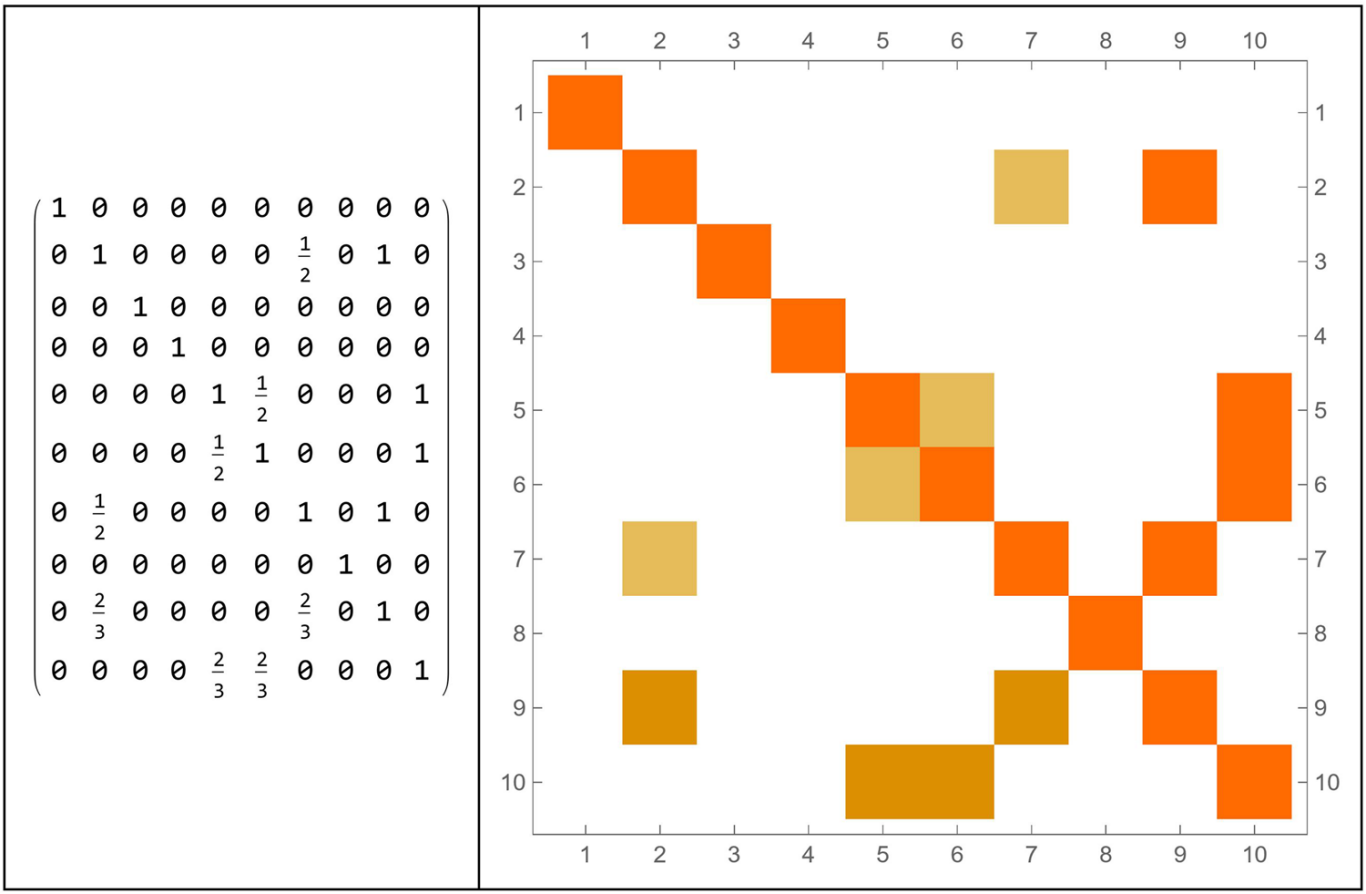
Intersection array and correlation matrix for representing relationships among the emerged circuits.

A different way to represent this information is in the form of an intersecting array as follows (Fig. 9). Given two circuits $r$ and $s$, we can calculate the ratio between the number of elements ${i}_{rs}$ contained in the intersection between $r$ and $s$, divided by the number ${n}_{r}$ of elements of $r$. For example, ${i}_{\mathrm{2,7}}=\frac{1}{2}$ , while ${i}_{\mathrm{2,9}}=\frac{2}{2}=1$.

If we carefully analyze these circuits, we notice some interesting processes:

- The circuit 2, made by the Brodmann areas $\left\{{\text{5L}},{\text{43L}}\right\}$ is a subset of the circuit 9, made by the Brodmann areas $\left\{{\text{5L}},{\text{32L}},{\text{43L}}\right\};$
- The circuit 5, made by the Brodmann areas $\left\{{\text{24L}},{\text{26R}}\right\}$ is a subset of the circuit 10, made by the Brodmann areas $\{24\mathrm{L},26\mathrm{R},35\mathrm{L}\}$;
- The circuit 6, made by the Brodmann areas $\{24\mathrm{L},35\mathrm{L}\}$ is again a subset of the circuit 10, made by the Brodmann areas $\{24\mathrm{L},26\mathrm{R},35\mathrm{L}\}$;
- The circuit 7, made by the Brodmann areas $\{32\mathrm{L},43\mathrm{L}\}$ is again a subset of the circuit 9, made by the Brodmann areas $\{5\mathrm{L},32\mathrm{L},43\mathrm{L}\}$;
- Both Brodmann Areas 43L and 24L are in common with 3 circuits;
- Brodmann areas 5L, 5L, 32L and 26R are in common with 2 circuits.

Regarding points (a)–(d), it means that the pairs of areas are strongly related to each other, while the third area of each circuit is weakly correlated.

### The emergence of circuits

**Figure 10:**
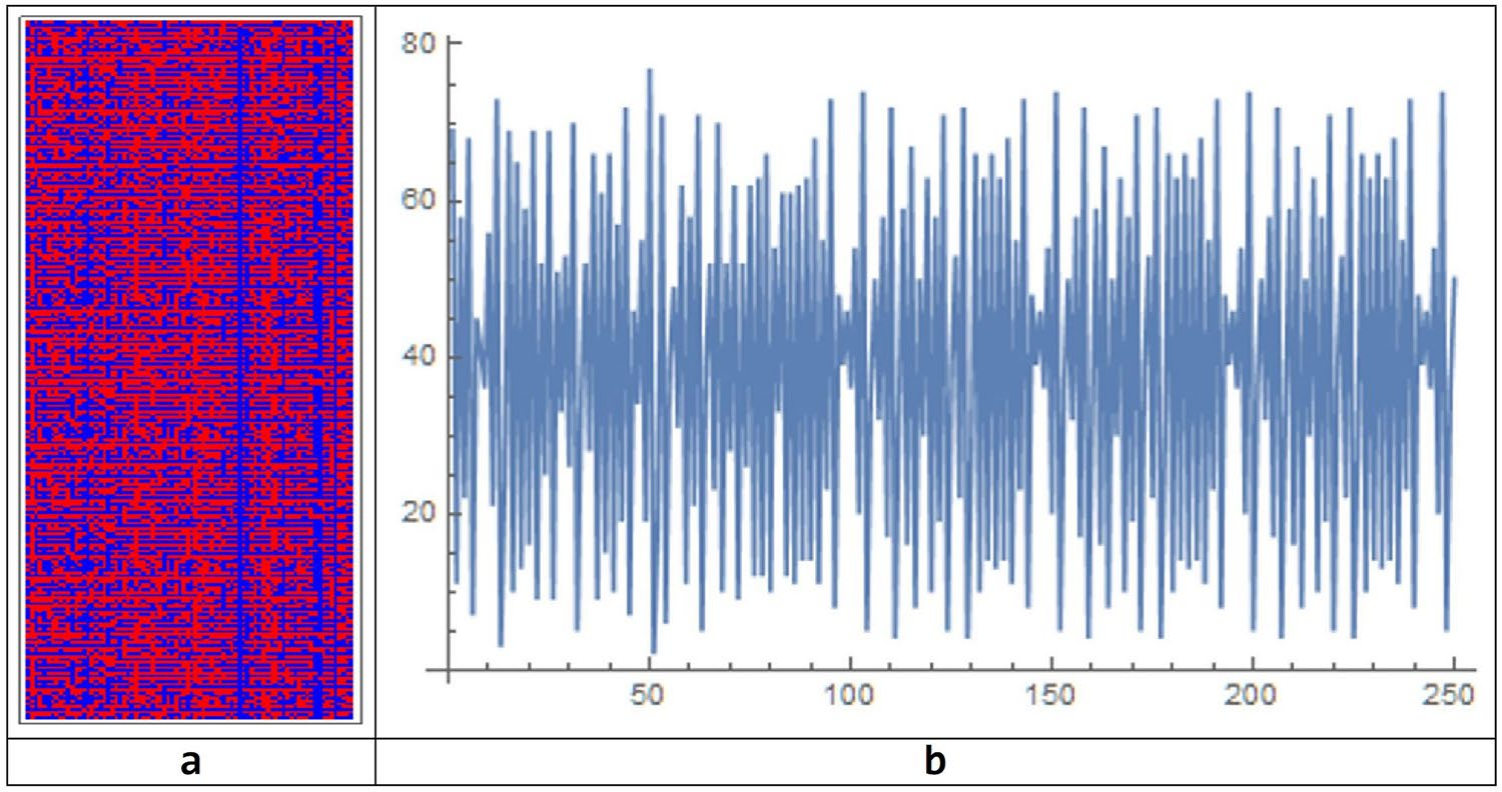
Spatio-temporal pattern of evolution of the BN system chosen as example after 250 simulation steps (**a**), together with the time-series of the evolution showing the periodic behavior of the system (**b**).

Using the simulation chosen as an example, and starting from the 16 active brain areas identified for the Action–Execution cognitive process, taken as initial data, let us analyze how the circuits emerge along the evolution of the spatio-temporal structure. After 250 time steps, the system evolves with a periodic configuration (Fig. 10).

At step 89, there are 63 active areas: {1L, 2L, 2R, 3L, 3R, 4L, 4R, 5L, 5R, 6L, 6R, 8L, 8R, 9L, 9R, 11L, 11R, 17L, 17R, 18L, 18R, 19L, 20L, 20R, 21L, 22L, 22R, 23L, 23R, 24L, 25L, 25R, 26R, 28L, 28R, 29L, 30R, 32L, 32R, 34L, 34R, 35L, 35R, 36R, 37L, 37R, 38L, 39R, 40L, 41L, 41R, 42L, 42R, 43L, 43R, 44L, 44R, 45L, 45R, 46L, 46R, 48L, 48R}. From this moment, the system went on an oscillating attractor of period 48 and repeats an endlessly periodic pattern: the ten previously-mentioned circuits emerge in their final organization.

After 50 time steps, we observe the emergence of the following 9 circuits: {5R, 45L}, {18L, 22R}, {20R, 23R}, {24L, 34L}, {24L, 35L}, {27R, 36L}, {34R, 46R}, {42R, 45R}, {24L, 34L, 35L}.

Of these, only 2 {24L, 35L} and {R 42, R 45} are in common with the final circuits. We may think that these circuits, once they are formed remain even later. In reality it is not so. In fact, if we analyze the spatial and temporal pattern between 50 and 100, we observe the formation of more than 16 circuits that are: {2R, 38L}, {17L, 20R}, {20R, 23R}, {22L, 48L}, {24R, 36R}, {26R, 45R}, {2R, 4L, 20L}, {2R, 24R, 36R}, {2R, 28L, 28R}, {9R, 26R, 45R}, {28L, 28R, 48L}, {2R, 4L, 9R, 45R}, {4L, 17L, 20R, 23R}, {4L, 22L, 28R, 48L}, {4L, 9R, 20L, 20R, 48L}, {2R, 9R, 20L, 28L, 36R, 38L}. Of these, only {17L, 20R} is equal to the final circuit. There is poor correspondence between the circuits of the transient regime with respect to the composition of the final circuits, when the simulation turns to the end, after going on a steady-state attractor. Occasionally the circuits of the transient regime contain an area of the steady-state circuits. In this sense, the transient behavior does not seem to prevent in any way what final circuit configuration will appear like at the steady state.

### The emergence of circuits at the local level

To analyze better the emerging rhythmic and oscillatory behaviors, let us now examine a different and more regular dynamic, compared to the more complex one in the previous example. Also with the parameters a = 1 and b = 4, we only consider the 16 active areas initially. After just 16 steps the system reaches the periodic attractor. The oscillation period is 6 time steps.

**Figure 11:**
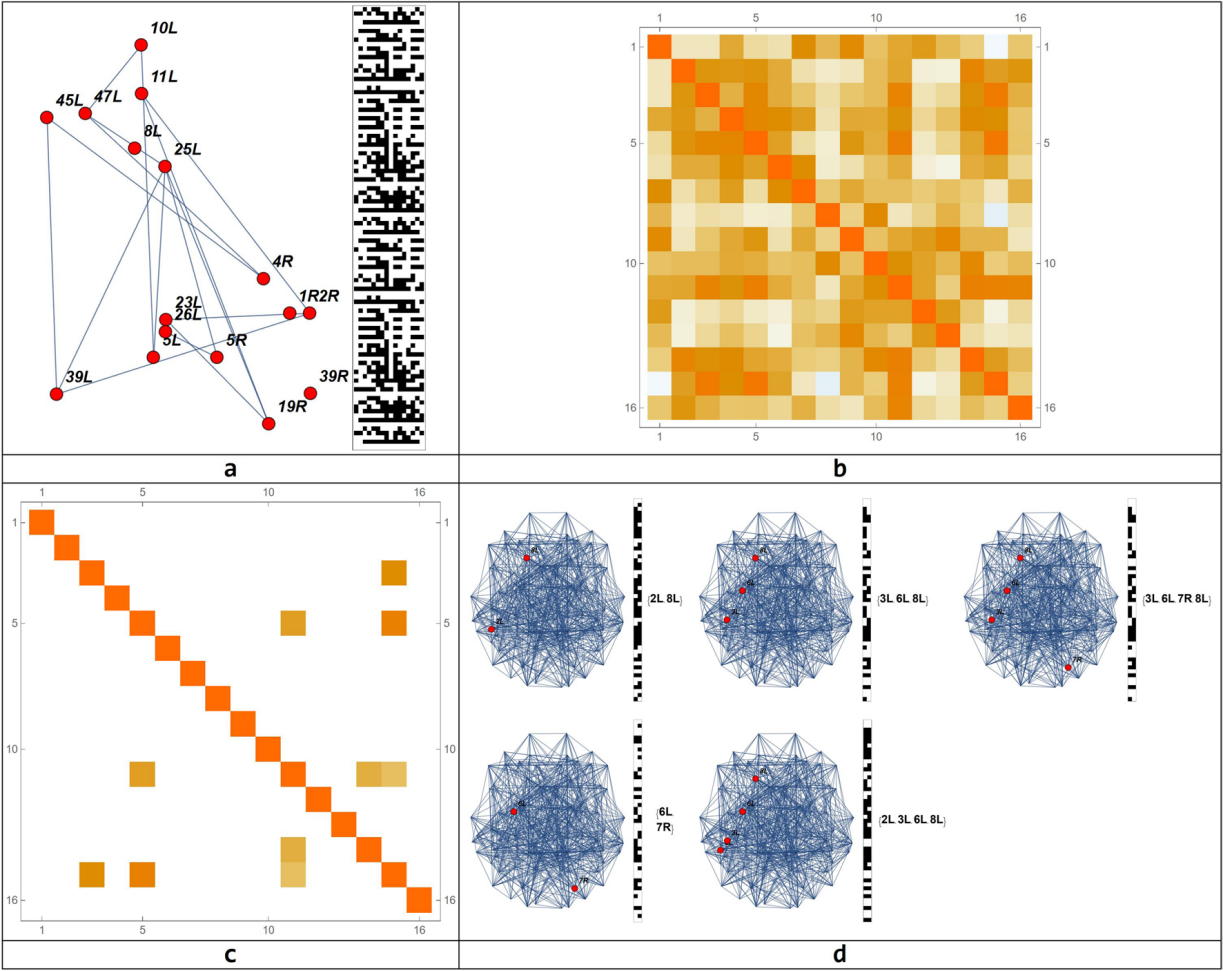
Representation of the Action–Execution circuit in the brain connectome made of 82 nodes, its connection matrix, the connection matrix of the emerging circuit and their connectome representation. (**a**) Circuits local evolution; (**b**) Connection matrix; (**c**) Connection matrix of the emerging circuits (**d**) Connectome representation of the emerging circuits.

Let us now analyze the circuit of Action–Execution as a distinctive one, as represented in Fig. 11, allowing its evolution at the local level of the BN, the realization of the connection matrix, the detection of its main circuits and lastly, their representation on the brain connectome.

To obtain the emerging circuits of this simulation, it was necessary to slow down the threshold of the connectivity ($z=0.6$), as a very high level of connections may hide the process (see Table 7).

**Table 7:** Circuits emerging from the Action–Execution evolution with a BN.

	Connectome nodes	Brodmann areas
Circuit 1	{4, 15}	{2L, 8L}
Circuit 2	{11, 14}	{6L, 8R}
Circuit 3	{5, 11, 15}	{3L, 6L, 8L}
Circuit 4	{3, 5, 11, 15}	{2L, 3L, 6L, 8L}
Circuit 5	{5, 11, 14, 15}	{3L, 6L, 7R, 8L}

We hypothesize that these circuits represent the firing over time of all areas involved in the simulated process, with oscillations between areas.

### The dynamics of the emerging circuits: stationary, oscillatory and rhythmical behavior

To better understand the nature of these emergent circuits, let us examine another case in which $a=1$ e $b=6$ maintaining the same threshold $x=0.9$. Results about the evolution of the Action–Execution circuit and the relationships that each area engages with the other ones by means of the phenomenon of binding together by symmetry^80^ are presented in what follows. We have identified oscillatory, stationary and rhythmical repetition of dynamical behavior for the emerging networks. Nodes always switched on or off are as follows $: \{\mathrm{1,3},\mathrm{5,9},\mathrm{12,15,16,21,24,35,54,56,65,73,74,78,82}\}$.

**Figure 12:**
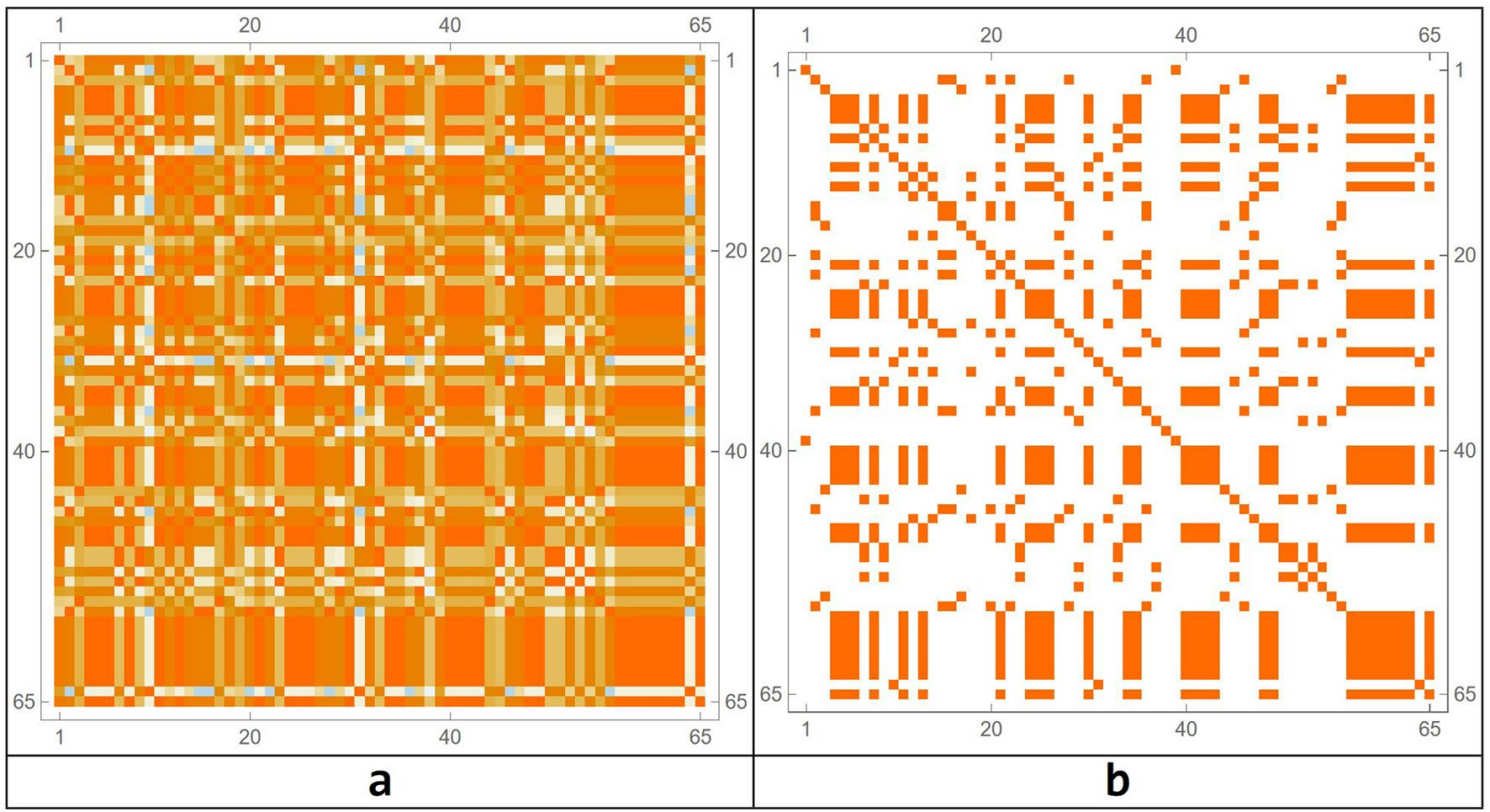
Correlation matrices for all nodes (**a**) and for the emerging circuits (**b**).

The connection matrix for those nodes that vary in time is represented in Fig. 12, together with the connection matrix for circuits.

The patterns of dynamic behavior are reported in Table 8.

**Table 8:** Emerging circuits and their dynamical behavior with $\mathrm{a}=1$ e $\mathrm{b}=6$ maintaining the same threshold $\mathrm{x}=0.9$.

	Connectome nodes	Dynamics of each circuit	Explanation
Circuit 1	$\{\mathrm{2,49}\}$		In this pattern of behavior, this neural circuit realizes the following performances: first nodes turn on, then they go off. Later, one turns on, then both turn off. Hereafter, the circuits travel by synchrony (all on or all off), until the cycle is stopped
Circuit 2	$\{\mathrm{17,41}, 80\}$		In this circuit, made of 3 nodes, the oscillatory behavior is very simple going from all nodes turned off for 2 steps and then all nodes turned on. Then there is an alternation between three steps with all nodes turned off and two steps with all nodes turned off
Circuit 3	$\{\mathrm{6,26},\mathrm{ 55,68}\}$		In this circuit, at each 5 steps, all 4 nodes rythmically turn off, then they turn on for one step
Circuit 4	$\{\mathrm{38,47},\mathrm{ 64,67}\}$		In this circuit, at each 3 steps all 4 nodes rythmically turn off, then they turn on and off for two steps
Circuit 5	$\{\mathrm{19,22,27,37,42,59}\}$		This circuit has the same dynamics as circuit 4, with a different number of nodes
Circuit 6	$\{\mathrm{11,14,32,43,57,62,63,66}\}$		In this circuit, after one steps of all nodes turned on and one turned off, it follows two steps of all nodes turned on and off
Circuit 7	$\{\mathrm{4,23,25,29,31,38,46,58,69}\}$		In this circuit, after two steps of all nodes turned on and off, it follows three steps of all nodes turned on
Circuit 8	$\{\mathrm{7,8},\mathrm{10,13,18,20,30,33,34,36,40,44,45},$ $\mathrm{50,51,52,53,60,61,70},\mathrm{71,72,75,76,77,79,81}\}$		This is a very simple circuit with a large number of nodes which rythmically turn on and off at each step of the simulation

Table 8, related to the previous parameters, shows circuits exhibiting periodic behaviors with low periods, even in the presence of a larger number of involved areas. This means that widening the activation range ($b-a$) leads the behavior of the circuits to greater regularity***.***

### The emergence of circuits at the global level

By modeling the brain dynamics with Boolean networks, we detected the emergence of cognitive circuits, which are very large-scale-networks. From the simulations carried out extensively for all system parameters, we found many circuits. Usually, the circuits emerge quickly, in large numbers. For the chosen reference, we found that 9 circuits emerged from 0 to 50 simulation steps. Of these, two are in common with the circuits detected at the end of the simulation. 16 circuits emerged between 50 and 100 simulation steps, but only 1 is in common with the circuits detected at the end of the simulation. Subsets of circuits that emerged at the end of the simulation are related to the huge circuits emerged at the beginning of the simulation.

**Figure 13:**
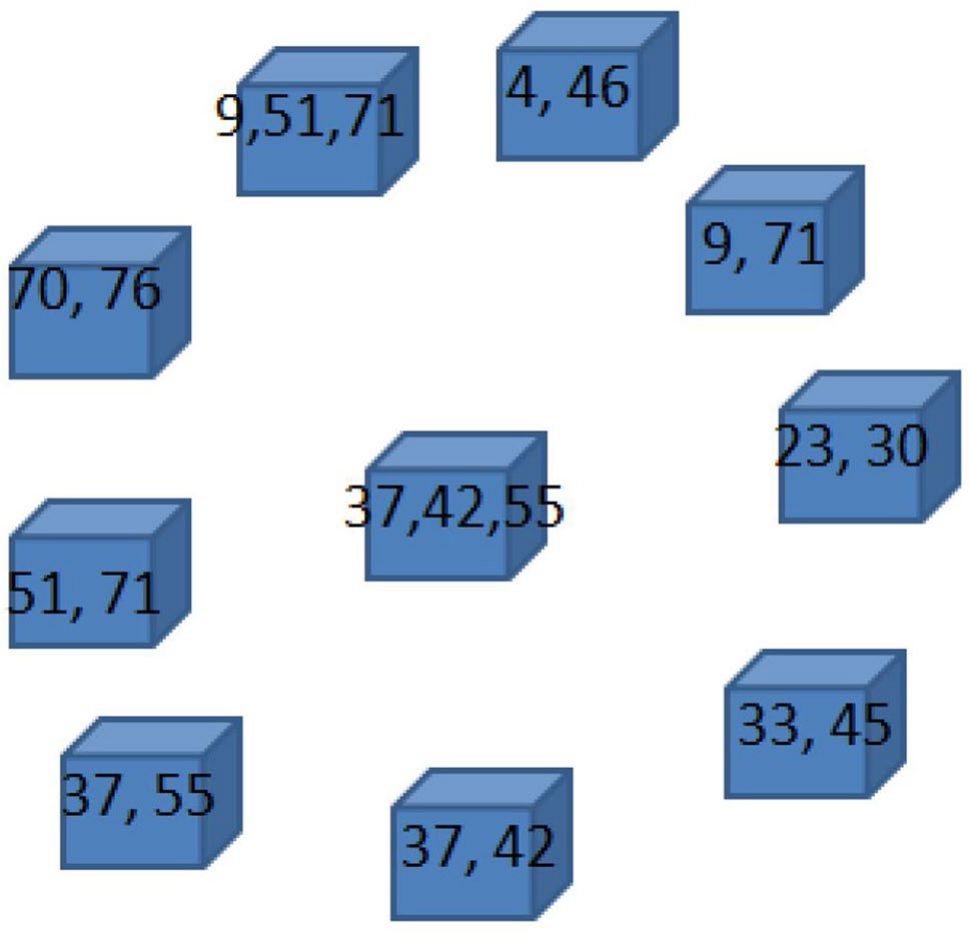
The 10 circuits, obtained from the NB simulation taken as model, are compared to other circuits which could emerge from different simulation parameters.

Let us try to analyze the results on the BN global dynamics behavior using the same simulation as an example. Considering $$a=1$$, $$b=4$$, ($$x>0.9)$$ as network parameters and threshold values respectively, we got 10 circuits (Fig. 13), considered as a reference configuration to which to compare all the other configurations, obtained by changing the parameters $$a$$, $$b$$ and $$x$$. This circuit configuration was chosen because it is very close to the physical organization of the behavioral map, drawn from the ICAs standard. It could be represented also in the brain and it is realistic from a cognitive point of view as a computational model.

In our case, we limited our attention to the understanding of what is going on in the cognitive circuits stability, taking this first circuit configuration as an example and comparing all the circuits obtained by varying the parameters to this reference frame, changing one parameter at a time.

Our hypothesis is that such circuits can vary slightly within certain limits. So, not all those that vary greatly, can be considered believable from the point of view of their cognitive reliability.

## Influence of the connection threshold on the stability of the emerging circuits

Let us change the Boolean Network threshold for the following values of the $x$ parameter:

$$x = \mathrm{0.89,0.90,0.91}$$

**Figure 14:**
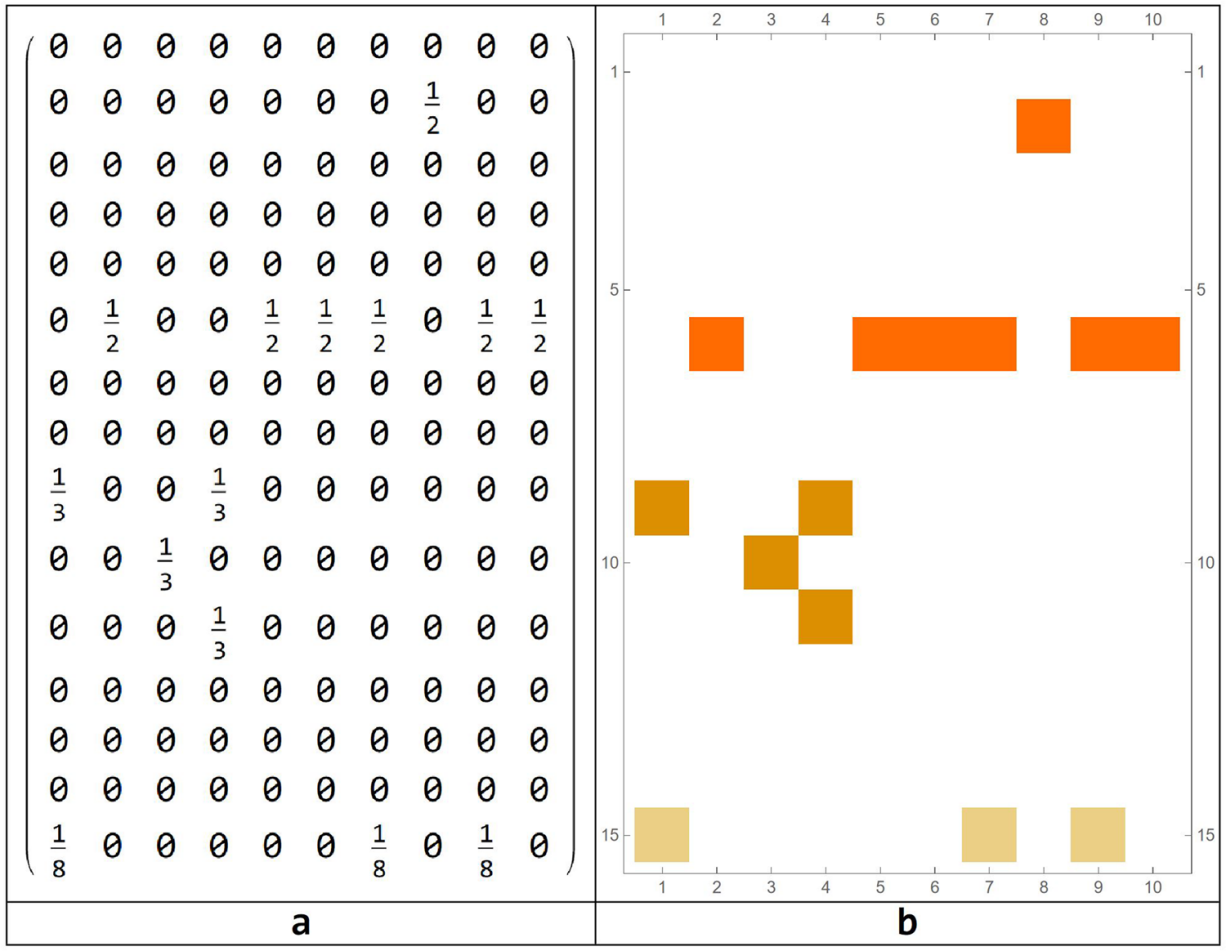
Intersection array (**a**) and correlation matrix for different simulation parameters (**b**).

Unlike the case considered as reference with $x = 0.90$, for which we obtained 10 circuits, for $x = 0.89,$ 15 circuits emerged from the simulation run. To evaluate the possible overlapping among the two different simulation runs, and consequently among the two sets of emerging circuits, an intersection between all the possible circuits of the two simulations has been done. We can represent these intersections using the same method of Fig. 9, obtaining a matrix shown in Fig. 14.

As can be seen, there is no relationship between the two groups of circuits. However, it is interesting to observe that, as occurred at the local level, a new circuit, composed of two areas $\left\{{\text{24L}}, \, {\text{43L}}\right\},$ appears. These two areas already had an important role in the simulation, taken as a reference model. In fact, the new emerging circuit has at least one element in common with six circuits of the reference case. Instead, considering a threshold level of $x = 0.91$, we observe the emergence of 22 circuits, of length: $\{2,2,2,2,2,3,4,5,6,6,7,9,11,12,12,13,13,15,15,16,17,19 \}$.

**Figure 15:**
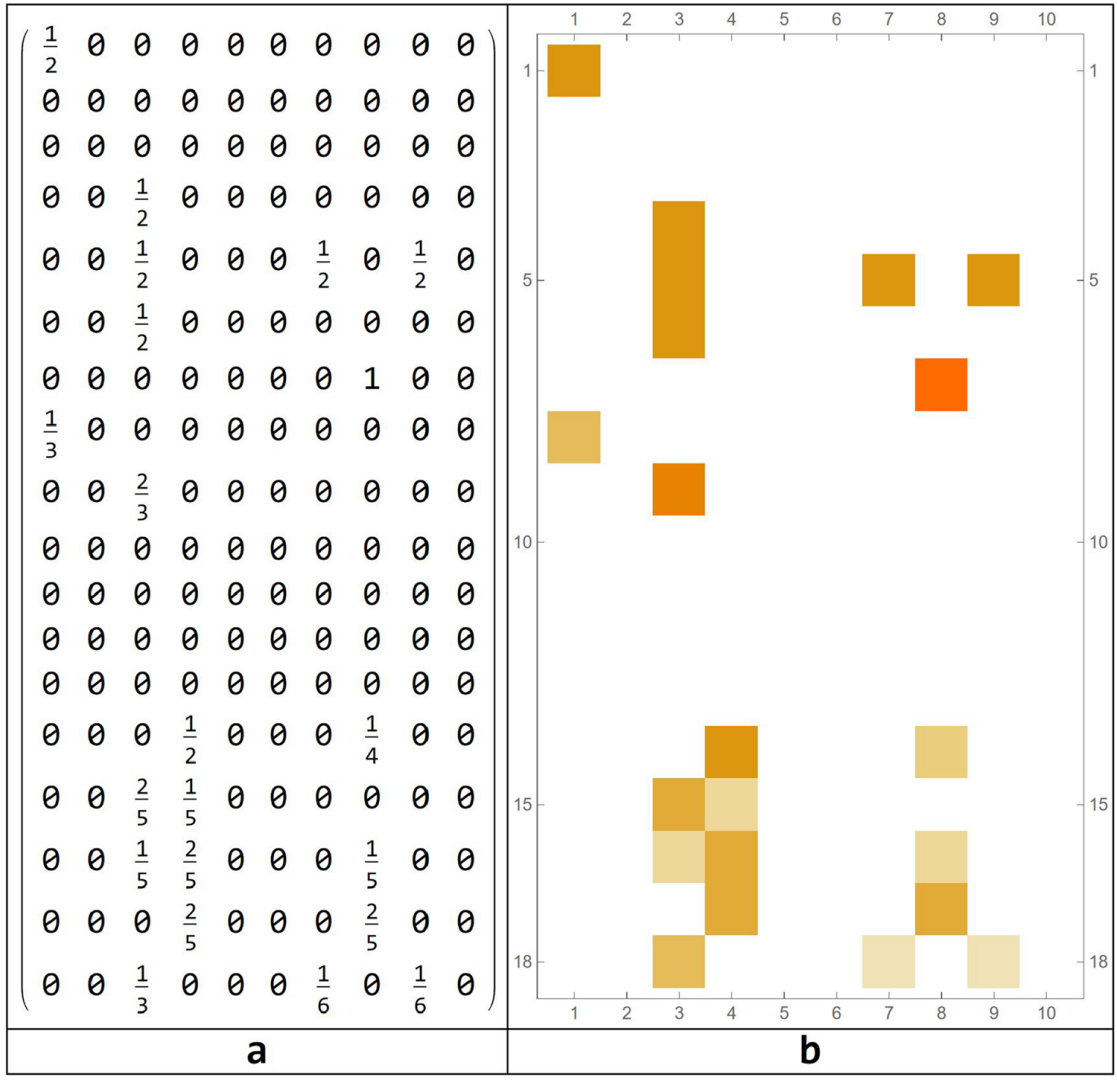
Intersection array (**a**) and correlation matrix for different simulation parameters (**b**).

Therefore, in addition to the presence of small circuits made up of at least of three elements, we discovered the presence of larger circuits, composed of many elements. The relationships between these new set of circuits and the circuits emerged for the reference model simulation, are graphically expressed as follows (Fig. 15).

Circuit 1 (repeated 3 times) and circuit 3 (contained in 5 of the new circuits), continue to exist as sub-circuits of larger circuits. So, the threshold according to which the Boolean network is built changes the topology of the systems and it is critical for the definition of the emerging circuits, which, in turn, change significantly, even for small changes of this threshold.

In this sense, the circuits are not stable in relation to the network topology, where even small variations can trigger dramatic changes in the definition of these circuits.

### Variation of parameters ${\varvec{b}}$ and ${\varvec{a}}$

Let us analyze what happens varying $b$ compared to the reference value of $b = 4$. In the case where $b = 3$, only one circuit emerges, composed of the 9R and 23R Brodmann areas This circuit has no area in common with the reference example circuits. In the case where $b = 5$, the process became more interesting, with 18 circuits, of length {2, 2, 2, 2, 2, 2, 2, 3, 3, 3, 3, 4, 4, 4, 5, 5, 5, 6}. The overlap of these circuits with the reference ones is fairly low. In this case, circuit 3 of the reference model, consisting of areas {17L, 20R} is contained in three new circuits, of larger size. The reference circuit 8, composed of the Brodmann areas {42R, 45R}, remained as a sub-circuit of a larger circuit.

In the case in which a = 2, no circuits appear and so no comparison is possible.

### Correlation threshold

The correlation matrices are used to identify circuits, analyzing the related areas. In particular, an area is related to another area if the correlation of the signals associated with the two areas is greater than a certain threshold $z$. Given an area $r$, and a threshold $z$, the set of all areas ${A}_{r}$ whose correlation with the $r$ area is greater than $z$ represents a circuit, adding $r$. Each circuit is counted only once. It is important to note that two areas ${a}_{1}$ and ${a}_{2}$ can belong to the same circuit, even if the correlation between them is less than $z$. This is the case of the reference circuits 2, 7 and 9. Circuits 2 and 7 have two areas correlated with $z> 0.87$. Their union represents a new circuit (circuit 9) that contains three elements. Two pairs, corresponding to circuit 2 and 7 have a higher correlation of $z$, while the third pair has a correlation $<z$. As $z$ decreases, the starting circuits are maintained, and maybe are incorporated in larger circuits, while for growing values $z$, these circuits instead tend to clear. The new circuits will be subsets of the starting circuits.

Let us analyze in detail different correlation thresholds.

For $z = 0.86$, there are still 10 circuits and obviously identical to those of the reference case. The case where $z = 0.85$ is more interesting where there are now 15 circuits of length {2, 2, 2, 2, 2, 2, 2, 2, 2, 2, 3, 3, 3, 3, 4, 4, 6}. It was possible to observe that the first circuit continued to subsist; circuit 2 continued to exist individually and was also part of a larger circuit. Even circuit 3 became part of a larger circuit. Circuit 4 continued to maintain its independence, while 5 appeared as a single entity and also part of a larger circuit. Let us observe, however, how the circuits are developed in the direction of increasing thresholds. If $z = 0.88$, there are now 8 circuits. All the reference circuits continue to survive, with the exception of circuit 5, which disappears, and circuit 10 that has lost one of the three areas coinciding with circuit 6. If we further increase the threshold to 0.89, we observe that now there are now 5 circuits as seen from the Figure xxu1. The only circuits that tend to survive are circuits 3, 4, 6, 7 and 8. While circuits 9 and 10 lost each area of the three they had previously. The result did not change until value z = 0.92; while for z = 0.93 the only circuit that continued to survive was 8, corresponding to the two Brodmann areas {42R, 45R}.

## Discussion and conclusions

To model structural and functional aspects of brain dynamics better and to be able to grasp cognitive behavior, we used Boolean Networks for the following reasons:

- The attractiveness and ease-of-use of the BN model^81^ which, by simplifying the complexity of brain dynamics, allows the emergence of circuits and chain of circuits (the network’s attractors ) which in turn represent stable (or metastable) sets of states of the brain toward which the system converges. Therefore, evolving toward attractors means to reach a specific behavioral goal (probably by a completely mechanical internal dynamics). This approach helps to predict the brain’s organization at different scales (the single circuit dynamics at the level of each brain area, the emergence of circuits that carry out the specific functional task, the completely new emergence of circuits, which could shed light on the long-range informational process in the brain). In this way, we have a running idea of what is a cognitive-functional attractor and what is the structural side of the system that allows its dynamics.
- Current understanding of large-scale-cognitive-networks is incomplete^3,82,83^. Therefore, comparing simulated dynamics with observed brain networks of a certain cognitive behavior may help in evaluating their reliability in predicting and showing missed information which could not be seen, by using real life methods. BN helps in predicting the process, picking up the qualitative and quantitative changes in the dynamic expression of the cognitive behavior.
- This model allows observation of the structure and lengths of the behavior, especially when expressed in recurrent patterns of activation. This in turn allows the determination of the basins of attraction of the brain circuitry, and to analyze the flow of information through the brain, thus determining the phase transition between chaotic, transient, ordered patterns, also discovering the edge of chaos^84–86^, which is where complex computation occurs. In addition, topology is of primary importance, owing to the fact that different dynamic behavior and flow of information can occur according to different parameter values, initial conditions, thus giving account of the brain as a dynamic non-linear^87^ entity.
- BN well fits with the anatomy of the brain, represented as a connectome, for understanding basic brain functioning, and, therefore, both the structural and functional sides of the brain can be properly replicated in a biological-like structure. The finite set of the activation rules and updating scheme of the BN, together with the possibility of varying the synchronous or asynchronous method, and the initial conditions allow a reduction of the potentially infinite number of processes a brain can carry out. For each node, the activation rules are determined by the activation states of the other nodes in the previous transition step, synchronously or asynchronously. These critical features of the BN dramatically influence the number and characteristics of the attractors. As brain areas activation rules and neighboring interaction among areas are not known, by using BN different activation rules and schemes can be tested, which best fit with conditions under which the simulated network would be able to capture the observed cognitive processes.

Another point of discussion is the choice of different parameter values of the NB, especially for threshold values. The threshold became the filter to see how nodes of the brain are individually correlated with the other nodes. It was crucial to establish the parameters according to careful neurocognitive modeling. This process would allow understanding the relationships between brain areas that are activated for the cognitive process we are describing. This, however, would require that it can be known, a priori, what these connections are from the neurophysiological point of view. As in most cases there is no data in this context, we tried to do some evaluations of the best possible parameters. We can state that the rule of evolution of the Boolean network is crucial to the stability of the brain circuits, which, therefore, significantly change their characteristics. For increasing values of $b$ and decreasing values of $b$, we observed the declining of many circuits, while for growing values of $b$ many circuits disappeared, although a few of them remained as subcircuits of larger circuits. For the simulations we realized, putting the threshold at 0.9, the system produced no important interactions, it did not reveal any meaningful structure. This meant that, the nodes of the brain circuitry having very strong connections, did not imply having effective interactions or correlations that can emerge. Placing the correlation threshold at 0.80, interaction among areas and many brain circuits emerged. We chose to put the correlation threshold at 0.87 in order to get a rich variety of connections. This question is strictly related to the brain local and global dynamics, and the observation of phenomena at different scales. According to this issue, we noticed mixed phenomena. The effects of the threshold levels changed the results of the simulations. In fact, if a very low threshold level allowed the emergence of the circuits at the local level, at the global level, this has not happened. This differs from the previously-argued hypothesis at the global level, for which we have shown that the average low threshold reveals an acceptable number of circuits. On the contrary, the high threshold increases the number of circuits. The phenomenon is certainly linked to the scale of observation of the dynamics that are emerging. Therefore, it can be stated that, according to the level of scale, the functions of the thresholds can vary, providing results of completely different brain circuital connections (although they can be related). So, at the global level, a low threshold can make the circuits disappear, while at the local one, they continue to function. From the mathematical point of view, we are dealing with two different measures, in respect of which, there is no correlation. If we were to use the global threshold for the local level, there would be no local circuits. Lowering the local threshold is as if we accept the existence of local weak signals and strong global signals. It is a modeling outcome. We do not know if it also has a corresponding phenomenon in neuronal areas at different scales. In fact, the correlation that we use is a coarse modeling, interesting at the global level, but less significant at the local one.

In conclusion, we can say that modeling with Boolean Networks makes sense from the neurocognitive perspective. In fact, in the cognitive process we have simulated, it is known that the first circuit affects the somatosensory cortex and the entorhinal cortex. This means that the two areas are activated for bringing spatial, visual and tactile information to the cognitive system (from the somatosensory cortex), which relates in some way to the stimulus localization. Then stimuli are conveyed to the entorhinal cortex system, where the grid cell systems provide information on the agent’s localization in the space in which he/she is in, especially thanks to the head position sensors, in correlation with a series of visual and auditory stimuli. These data, in turn, allow information to be obtained on the shape and size of objects on which he/she has to actually carry out the action. Furthermore, the entorhinal cortex connects with other systems, including the hippocampus, which plays a significant role for storing processes and actions that have already been realized. Brodmann area 43 L is common to circuits 2, 7 and 9, while Brodmann area 24L is common to circuits 5, 6 and 10. This might suggest that there are 6 circuit clusters of a higher level: the first four coincide with circuits 1, 3, 4 and 8, connected to Brodmann areas which do not communicate with other circuits. Two cerebellar areas are made first, from circuits 2, 7, 9, while the second of 5, 6, 10 circuits. In reality, circuit 9 has the two sub-circuits 2 and 7 whose areas are strongly correlated with each other via area 43L, while the other 2 areas are weakly correlated. Similarly, for circuit 10, which contains both circuits 5 and 6, weakly connected between each other and strongly by Brodmann area 24L.

A complex landscape of how cognitive processes emerge is given by the phenomenon we interpreted as the emergence of metastable structures, which gave computational data on large-scale networks emergence and on how the brain creates structures of remote communication. This is a metastable structure^88^, with a hierarchical organization, where each level allows for the emergence of brain organizations which behave at the next superior level.

In the dynamics of evolution for this simulation, we observed both stationary and oscillatory behavior, and repetitions of rhythms that can be ascribed to the different ways brain areas have to interact, in the Action–Execution simulation process. At the global level, after evaluating the whole period of the network, we denote that the above-described dynamics achieve their behavior each 48 steps of simulation, with complex organization at the edge of chaos. This problem can be extensively studied only with a great deal of computational power. In our case, we limited our attention to the understanding of what's going on in the cognitive circuit stability, taking as example this first circuit configuration and comparing all the circuits obtained by varying the parameters to this reference frame, changing one parameter at a time.

## Supplementary Information

Supplementary Information 1.

Supplementary Table S1.

## Data Availability

The original contributions presented in the study are included in the article/supplementary material, further inquiries can be directed to the corresponding author/s.

## Code availability

The code and software used in the study are included in the article/supplementary material.
